# Darkfield and Fluorescence Macrovision of a Series of Large Images to Assess Anatomical and Chemical Tissue Variability in Whole Cross-Sections of Maize Stems

**DOI:** 10.3389/fpls.2021.792981

**Published:** 2021-12-14

**Authors:** Marie Berger, Marie-Françoise Devaux, David Legland, Cécile Barron, Benoit Delord, Fabienne Guillon

**Affiliations:** ^1^UR1268 BIA, INRAE, Nantes, France; ^2^Limagrain Europe, Saint-Beauzire, France; ^3^PROBE Research Infrastructure, BIBS Facility, INRAE, Nantes, France; ^4^IATE, Univ Montpellier, INRAE, Institut Agro, Montpellier, France

**Keywords:** autofluorescence multispectral imaging, darkfield imaging, quantitative histology, macrovision, maize stem

## Abstract

The proportion and composition of plant tissues in maize stems vary with genotype and agroclimatic factors and may impact the final biomass use. In this manuscript, we propose a quantitative histology approach without any section labelling to estimate the proportion of different tissues in maize stem sections as well as their chemical characteristics. Macroscopic imaging was chosen to observe the entire section of a stem. Darkfield illumination was retained to visualise the whole stem cellular structure. Multispectral autofluorescence images were acquired to detect cell wall phenolic compounds after UV and visible excitations. Image analysis was implemented to extract morphological features and autofluorescence pseudospectra. By assimilating the internode to a cylinder, the relative proportions of tissues in the internode were estimated from their relative areas in the sections. The approach was applied to study a series of 14 maize inbred lines. Considerable variability was revealed among the 14 inbred lines for both anatomical and chemical traits. The most discriminant morphological descriptors were the relative amount of rind and parenchyma tissues together with the density and size of the individual bundles, the area of stem and the parenchyma cell diameter. The rind, as the most lignified tissue, showed strong visible-induced fluorescence which was line-dependant. The relative amount of para-coumaric acid was associated with the UV-induced fluorescence intensity in the rind and in the parenchyma near the rind, while ferulic acid amount was significantly correlated mainly with the parenchyma near the rind. The correlation between lignin and the tissue pseudospectra showed that a global higher amount of lignin resulted in a higher level of lignin fluorescence whatever the tissues. We demonstrated here the potential of darkfield and autofluorescence imaging coupled with image analysis to quantify histology of maize stem and highlight variability between different lines.

## Introduction

Maize is a major productive crop worldwide and the most widely used forage crop in dairy cow feeding (Boon et al., 2008; Barros-Rios et al., 2012; Barriere, 2017). In addition, maize stems are considered one of the key lignocellulosic feedstocks to produce biofuels and other value-added chemicals (Carpita and McCann, 2008; Barrière et al., 2009b; Melati et al., 2019). Many of these uses involve efficient degradation of the cell walls either by enzymes or microbes.

The cell wall in maize stems is a complex polymer network of polysaccharides, namely, cellulose, hemicelluloses, and phenolics, as well as other minor compounds, such as pectins and structural proteins (Carpita and McCann, 2008). Cell wall phenolics comprise lignins and hydroxycinnamates and para-coumaric and ferulic acid derivatives. Lignin is a heterogeneous mixture of randomly polymerised phenolic monolignols (Vanholme et al., 2019). In maize stems, the amount of lignin in the cell wall fraction typically ranges between 12 and 20% (Jung and Buxton, 1994; Méchin et al., 2000; Barrière et al., 2009a,b). Para-coumaric acid, which accounts for approximately 1.5–2.5% of the cell wall, is mainly associated with lignin, while ferulic acid, which accounts for approximately 0.7% of the cell wall, is ether linked to lignin or ester linked to hemicelluloses (Ralph et al., 1998; Méchin et al., 2000; Jung and Casler, 2006a; Barrière et al., 2009a; Hatfield et al., 2017). Ferulic acid can mediate cross-linkages between hemicelluloses and lignins or between hemicellulosic chains *via* diferulic bridges (Ralph et al., 1998; Hatfield et al., 2017; Terrett and Dupree, 2019). The amount of lignins in the cell wall, their variable structure, and the cross-linkages between cell wall components have been suggested to have a variable depressive effect on cell wall polysaccharide degradation by enzymes or microorganisms (Méchin et al., 2000; Fontaine et al., 2003; Jung and Phillips, 2010; Barriere, 2017; Casler and Jung, 2017; Hatfield et al., 2017; Terrett and Dupree, 2019). This effect is related to the limited access of enzymes or microbes to degradable/fermentable polysaccharides (Meng and Ragauskas, 2014; Melati et al., 2019). However, the main determinant among these factors for recalcitrance is still not clear (McCann and Carpita, 2015; Melati et al., 2019; Zoghlami and Paës, 2019), which is partly due to the large variability of biomass and assignment of biomass as a bulk material without considering the heterogeneity of plant cell walls according to organs and tissues.

In fact, within a species, the cell wall composition depends on the genotypes and the plant-breeding environment but also on other components, such as the organs, stems, and leaves to the tissues and cell types. For example, a maize stem or internode is composed of different tissues, namely, rind, parenchyma and vascular bundles, whose proportions, morphologies and compositions vary according to the genotype, maturity and agro-climatic conditions (Cone and Engels, 1993; Morrison et al., 1998; Jung and Casler, 2006a,b; Legland et al., 2017; Perrier et al., 2017; El Hage et al., 2018; Zhang et al., 2019). It has been shown that these tissues differ in their fermentation/digestibility yield and rate, which has been related to their cell wall composition (Akin, 1989; Scobbie et al., 1993; Wilson et al., 1993; Wilson and Mertens, 1995; Hatfield et al., 1999; Jung and Casler, 2006b; Barros-Rios et al., 2012; Ding et al., 2012; Devaux et al., 2018). Several authors have reported that the relative proportion of tissues and lignin distribution within organs can explain the differences in digestibility observed at an equivalent stage of maturity (Akin, 1989; Wilson and Mertens, 1995; Méchin et al., 2005; Barros-Rios et al., 2012).

To study the histological features of plant organs, methods are required to quantify the proportion of tissues and their composition. Microscopic techniques are generally proposed for this purpose. However, these methods are not compatible with large-scale or high-throughput studies. In the case of maize stems, the stem section area can be of 1–2 cm^2^ while the cells diameter can be of approximately 60 μm (Zhang et al., 2013; Legland et al., 2014, 2017); moreover, the experiments often need to repeated to tackle the biological variability. For a few years, whole stem section imaging has been developed (Zhang et al., 2013, 2019; Legland et al., 2014, 2017, 2020; Heckwolf et al., 2015; Perrier et al., 2017). Images of hand- or microtome-cut stem cross-sections were acquired with either a macroscope, a microscope slide scanner or a flatbed scanner. Different modes of illumination (darkfield, brightfield, and epifluorescence, etc.) associated or not with contrast-enhancing methods, such as Fasga staining (Tolivia and Tolivia, 1987), are implemented to visualise the tissues. Other authors favour 3D imaging and use micro-computed tomography technology for stem imaging (Zhang et al., 2018, 2020, 2021). Optical macrovision systems have the advantage of being relatively inexpensive compared to more sophisticated equipment, such as X-ray tomographs and are well suited for studying histology because they combine a large field of view and good spatial resolution, thus allowing for observations of a whole stem cross-section and differentiation of the different tissues (Legland et al., 2014; Corcel et al., 2016).

Regardless of the image acquisition methods, image analysis is required to identify and quantify morphological features, which are also called anatomical traits. Maize stems include the proportions of tissues, e.g., rind, parenchyma cells, and vascular bundles, and the morphology and density of cells and vascular bundles. Different image analysis workflows have been proposed, which depend on the targets and on the contrast in the images. Most workflows include a tissue segmentation step followed by morphological feature quantification. Heckwolf et al. (2015) developed custom image processing software that utilises a variety of global thresholding and local filtering to extract rind, pith and vascular bundle sizes from stem cross-section scanned images. Legland et al. (2017) proposed a series of morphological filters to identify the rind and vascular bundles in the pith from stem cross-sections after Fasga staining. Zhang et al. (2018, 2020, 2021) presented an image analysis pipeline to extract micro-phenotypic traits from 3D tomography images that combine threshold-based segmentation and morphological operations.

Once tissues are segmented, it is a straightforward process to measure the rind thickness, pith area, vascular bundle area or vascular bundle size or shape. These descriptors were related to stem lodging (Zhang et al., 2018), developmental stages (Zhang et al., 2020), and water stress (Legland et al., 2017; El Hage et al., 2018) or used to analyse the phenotypic variation between lines (El Hage et al., 2018; Zhang et al., 2021). In addition to tissue segmentation, Devaux and Legland (2014) proposed applying grey-level granulometry using morphological closings to directly extract cell size distributions from grey-level images. Legland et al. (2020) used the method on maize stem images to compute the parametric maps of cell size.

Chemical imaging techniques are required to reveal the variations in the cell wall composition of tissues or cell types. Specific staining methods or spectral imaging can be used for this purpose, and each technique leads to very different image analyses. Several authors have used Fasga staining to assess the distribution of lignin in maize or sorghum stem tissue according to developmental stages (Zhang et al., 2013, 2019) or in response to water deficit (Legland et al., 2017; Perrier et al., 2017; El Hage et al., 2018). Fasga staining colours lignified tissues in red and non-lignified tissues in blue. In Zhang et al. (2013, 2019), lignification was assessed by the ratio of red to blue intensity. The image analysis workflow made it possible to assess the distribution of lignin within a cross-section by profiling the red/blue intensity ratio from the epidermis to the centre of the cross-section. The image processing workflow was further improved and fully automated by Legland et al. (2017), and it was designed for measuring the amount of blue and red intensities in the parenchyma and the amount of red intensity in the rind. Perrier et al. (2017) also developed a dedicated tool in ImageJ software for analysing Fasga-stained cross-sections from sorghum internodes. The dedicated script allowed quantification of the outer zone area in percentage of internode cross-section area, the percentage of sclerenchyma tissue in the outer zone, the percentage of nonlignified tissue in the central zone of the internode and the density of vascular bundles in the central zone.

Apart from histochemical staining, spectral imaging techniques have been proposed to perform chemical mapping of cell wall variations in wood or plant stems. Microspectroscopy, such as Fourier transform infrared (FT-IR) or Raman microspectroscopy, is very useful to study carbohydrates or phenolic constituents (lignin or hydroxycinnamic acids) (Gierlinger, 2018; Beć et al., 2020). The main drawback is that the techniques are time-consuming, thus allowing for the mapping of only a small region of the sample, which limits the application of these techniques for the comparison of large numbers of samples. To monitor the chemical variation in tissue composition, multispectral fluorescence imaging can be applied (Corcel et al., 2016). Full-field fluorescence macroscopy has a sufficient spatial resolution (≈3 μm per pixel), high acquisition speed and large fields of view. Taking advantage of the autofluorescence properties of many plant compounds, fluorescence imaging can be performed with little tissue preparation and, more importantly, without labelling. Fluorescence imaging techniques have two main attributes over other techniques associated with their greater sensitivity and selectivity due to the unique properties of autofluorescent molecules being excited at a specific wavelength and emitting radiation at specific wavelengths. Plant cell wall autofluorescence is mainly linked to the presence of phenolic compounds, such as lignin and hydroxycinnamic acids. Hydroxycinnamic acids emit blue fluorescence under UV excitation at approximately 350 nm (Fulcher et al., 1971; Harris and Hartley, 1976; Lang et al., 1991), while lignin excited using UV and visible light emitted blue, green and red fluorescence (Djikanović et al., 2007; Donaldson et al., 2010; Donaldson, 2013, 2020; Donaldson and Williams, 2018). The nature of the phenolic compounds, their variable relative proportions and the environment (pH, presence of quenching molecules, etc.) result in variable tissue fluorescence responses that can be interpreted as a fluorescence tissue signature.

The analysis of multispectral images requires specific analysis tools that can account for both the spatial and the spectral dimensions of the image. Using the chemometric approach, the first step in the analysis of multispectral images is to process the spectral dimension of the data (Geladi and Grahn, 2006; Ghaffari et al., 2019). Spectral information can be extracted either manually or automatically from regions in the images (de Juan et al., 2009). In many cases, regions in the image are segmented based on the spectral information (Salzer and Siesler, 2014).

In this study, we developed a quantitative histology approach to estimate the proportion of different tissues in maize stem sections and associated a chemical profile with each of these tissues. Two macroscopic imaging techniques without prior labelling of the tissues were used. Darkfield macroscopy was chosen to visualise the different tissues independently of their chemical composition. In parallel, multispectral autofluorescence macroscopy was used to associate a multispectral autofluorescence profile to the tissues with the aim of evaluating the relative distribution of lignin and hydroxycinnamic acids. In the darkfield images, tissues and cells are visualised based on the diffraction properties of the light by the cell walls. An image analysis workflow was implemented to identify the tissues and then extract 2D morphological descriptors. We propose that a simple stem model can be used to estimate the volume descriptors of the amount of rind, vascular bundles and parenchyma cell walls. We sought to measure multispectral autofluorescence pseudospectra in each tissue. Tissues were also segmented from the multispectral images using a “*sum of intensities*” image and a workflow similar to that of the darkfield images. Because the parenchyma near the rind has been revealed to have specific enzymatic degradation properties (Jung and Casler, 2006b; Devaux et al., 2018), two regions of parenchyma were considered, and we evaluated and compared their fluorescence properties, i.e., relative amounts of lignin and hydroxycinnamic acid. Two stem internodes of 14 inbred lines were analysed with the aim of demonstrating the feasibility of the method suggested here. Correlations between the histological descriptors and the amounts of phenolic compounds and digestibility measured at the stem level for the 14 inbred lines were examined.

## Materials and Methods

### Plant Material and Stem Cell Wall Characterisation

#### Plant Material

Fourteen maize inbred lines selected for their contrasting digestibility were grown in Arras (France) in 2018. Twelve plants per inbred line were harvested at the silage stage. The stems were separated from leaves, panicles and ears. The internode located under the main ear was collected for two plants per line and stored in 70% ethanol/water (v/v) for quantitative histology. The stems of the remaining 10 plants were pooled, chopped and oven dried (70°C). The dried stems were ground with a hammer mill to pass through a 1 mm screen for the analysis of phenolic compounds and cell wall enzymatic digestibility.

#### Chemical Analysis

Cell wall material was prepared from the 10 dried and ground pooled stems. The ground material was placed in 80% ethanol at 100°C in an automated solvent extractor (ASE 350, Dionex Sunnyvale, CA, United States; 6 min flow time, 2 mL/min flow rate, 150% flush, and 30 s purge). The ethanol insoluble material was taken as the cell wall estimate (Chazal et al., 2014) and therefore called cell wall content and expressed in percent of the dry matter. Using the automated solvent extractor, the standard deviation is less than 1%.

The Klason lignin content was measured according to Dence (1992). Ester-linked para-coumaric and ferulic acids were measured after mild alkaline hydrolysis as described by Ho-Yue-Kuang et al. (2016). Analyses were performed in duplicate, and the results are expressed as the percentage of dry matter.

#### Digestibility Measurement

The enzymatic digestibility was measured in duplicate on the extractive-free material using the Aufrère and Michalet-Doreau method (Aufrère and Michalet-Doreau, 1983). The technique involves three stages: (1) pretreatment with pepsin (pepsin Merck 2000 FIP U/g Art7190) in hydrochloric acid (0.2% pepsin in 0.1 N HCl in a water bath at 40°C for 24 h; (2) starch hydrolysis in a water bath in the same mixture for *exactly* 30 min at 80°C; and (3) attack by cellulase (cellulase Onozuka R 10 extracted from *Trichoderma viride*, Yakult Honsha Co. Ltd, Japan, 1 g/L in 0.05 M sodium acetate buffer, pH 4.6) after filtration and rinsing for 24 h in a water bath at 40°C. The final residue was weighed. Due to the low starch content (<2% of the dry matter content of the alcohol-insoluble material), cell wall digestibility was equated with dry matter digestibility and calculated as follows:


(1) $$\text{IVCWD}=\frac{\text{M}1-\text{M}2}{\text{M}1}*100$$

where M1 is the dry mass of the extractive-free sample and M2 is the dry mass of the residue after enzymatic degradation.

### Image Acquisition

#### Sample Sectioning for Histological Analysis

For the two internodes retained for histology, a one cm long segment was sampled in the middle of the internode. For each segment, 150 μm thick cross-sections (called sections in the following) were cut in air with a gsl1 microtome (Design and production: Lucchinetti, Schenkung Dapples, Zurich, Switzerland) (Gärtner et al., 2014) and stored in 70% ethanol at 4°C until image acquisition. Prior to image acquisition, the sections were rehydrated in water overnight at 4°C to remove the air.

#### Darkfield Imaging

Images were acquired using the “BlueBox” macrovision acquisition prototype specially designed to observe plant tissue sections at the macroscopic scale without any prior labelling steps (Devaux et al., 2008, 2009). A monochrome CCD camera (Prosilica Digital Camera DCAM 1.31 – distributed by Alliance Vision, Montélimar, France) was equipped with a 1.2X magnification lens (Navitar Precise Eye, Rochester, NY, United States). With these settings, the images were 1,620 × 1,220 pixels and corresponded to a field of view of 5.92 × 4.43 mm^2^, with a pixel size of 3.63 μm. Grey levels were coded between 0 (black) and 255 (white). An optical fibre ring was connected to an intensity-controlled light source (SCHOTT DCRIV Light Source, Mainz, Germany) and placed under the samples to provide darkfield illumination. Motorised stages for positioning the camera and the samples allowed for the acquisition of large images. All elements were placed in a box to prevent outside light from entering. Homemade software developed under LabView was used for image acquisition.

**FIGURE 1:**
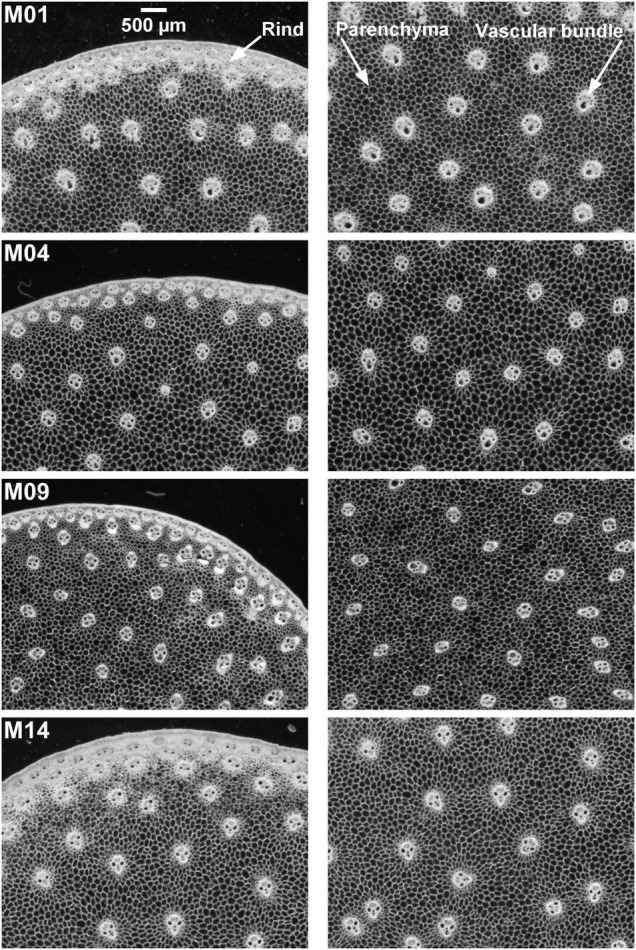
BlueBox images. Example of inbred lines: individual fields of view of cross-sections. Fields of view: 5.92 × 4.43 mm^2^. Rind, parenchyma cells, vascular bundles are visible. Rind thickness and vascular bundle and cells size vary according to the inbred lines.

**FIGURE 2:**
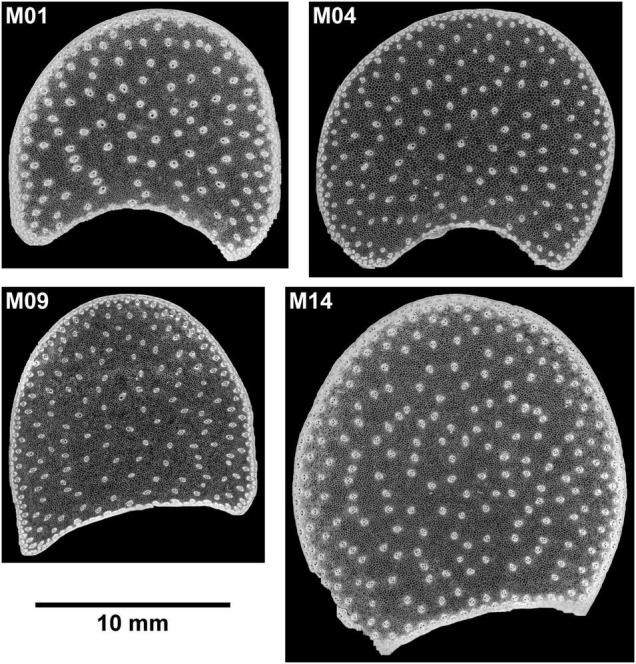
BlueBox images. Example of large images for four inbred lines. The large image reveals the stem section area, rind, vascular bundle repartition, and parenchyma.

Sections were placed between two round lamellae for observation. Mosaic images, called large images, were acquired to observe the entire sections. The largest images corresponded to a field of view of 20 × 20 mm^2^. Two sections per internode were imaged. Images of one internode were removed for M06 because air was still present after overnight rehydration. Finally, 54 large images were obtained. Examples of individual fields of view and large images can be seen in Figures 1, 2. Several tissues were observed within the stem sections: the rind, the vascular bundles and the pith parenchyma (Esau, 1977).

#### Multispectral Autofluorescence Imaging

Autofluorescence images were acquired using a Multizoom AZ100M fluorescence macroscope (Nikon, Japan) equipped with a Q Imaging EXI Aqua monochrome camera plus an RGB-HM-S-IR filter wheel for colour image acquisition. The system provides 1,392 × 1,040 pixel RGB images with grey-level intensities coded using 16,386 values. The total magnification was set to X4 by combining the AZ-Plan Fluor 2X lens (NA: 0.2/WD: 45 mm) and a X2 optical zoom. With these settings, the pixel size was 2.78 μm and the field of view was 3.9 × 2.9 mm^2^. The macroscope was equipped with a Prior Proscan II (Nikon, Japan) motorised stage, which allowed large image acquisition. The INTENSILIGHT (C-HGFI/C-HGFIE Precentred Fibre Illuminator Nikon, Japan) device with a mercury lamp ensured lighting for fluorescence imaging. Four fluorescence filter cubes corresponding to two UV excitations, namely, U1 and U2, and two visible excitations, namely, blue (BL) and green (GR), were placed inside the motorised filter wheel (Supplementary Table 1).

The acquisition software NIS-Elements (AR 5.02.02) allows automatised multispectral acquisition of large images. The multispectral sequence was designed to successively acquire the four RGB images corresponding to the four fluorescence filters for a given field of view before moving to the next field of view. The order of acquisition was GR, BL, U2, and U1, with exposure times set after viewing a few samples (Supplementary Table 1). After all acquisitions, the fluorescence intensity was found to be much lower for the two visible filters than for the UV filters, and a multiplicative factor of 2 was applied to the RGB images of the blue and green filters.

The resulting multispectral images contained 12 channels by merging the RGB images recorded using the four filter cubes (Corcel et al., 2016). The channels were put in an order from high to low wavelengths: blue, red and green channels of each RGB image acquired with filters U1, U2, blue and green. The channels names were U1b, U1g, U1r, U2b, U2g, U2r, BLb, BLg, BLr, GRb, GRg, and GRr. Channel U1r was removed from the sequence because it contained unwanted reflection from the excitation Rayleigh band. The final multispectral image therefore contained 11 channels.

For morphological image acquisition, rehydrated sections were placed in water between two round lamellae for observation. One multispectral image per internode was acquired (except for the anomalous M06 internode), and for seven inbred lines, a second section of one internode was imaged for repetition. The final set contained 34 large multispectral images.

### Image Analysis

Image analysis was performed in the MATLAB 2019b environment (Mathworks, Natick, MA, United States) using the image processing toolbox, dedicated homemade functions and scripts developed for BlueBox and macrofluorescence collections of images.

#### Image Representations

Displaying a set of large images is difficult, and the content of multispectral images cannot be viewed in a direct way. With the objective of comparing different inbred lines or sections and enabling details to be seen, a multiscale image representation was adopted. Zoom images corresponding to one field of view of the mosaic in the case of the BlueBox images were selected, and they showed details into the middle of the section and on the border of the section. Up to four large images with a resolution of 14 μm per pixel were compared. Low-resolution images (24 μm per pixel) were finally retained to draw A4 300 dpi figures, with one image per inbred line.

In parallel, an RGB representation of the multispectral fluorescence image was implemented. The red channel of the RGB image was computed as the average of the red channels U2r, BLr, and GRr. The green channel of the RGB image was computed as the average of the green channels U1g, U2g, and BLg. The blue channel of the RGB image was computed as the average of the blue channels U1b and U2b. The RGB images were called *composite macrofluorescence images* in the following. Two grey-level images were also computed for segmentation purposes: the image “sum of fluorescence intensity of the 11 channels” and the image “sum of visible fluorescence intensity” corresponding to the sum of the three channels BLg, BLr, and GRr.

#### Definition of Morphological and Autofluorescence Descriptors of Maize Stem Tissue

For each tissue, morphological and autofluorescence descriptors were defined. The stem area was retained as an absolute size descriptor. Relative areas were chosen to compare the rind, parenchyma and vascular bundle amounts. For the vascular bundles, the descriptors that were selected were the number density, individual surface area and elongation, which was defined as the width/length ratio. Parenchyma was also characterised by the cell size. Due to their different behaviour toward enzymatic degradation, two regions of parenchyma were considered (Jung and Casler, 2006b; Devaux et al., 2018): parenchyma near the rind and middle parenchyma. For each tissue, the average fluorescence properties were used to characterise the composition of cell walls. Measurements of the descriptors were performed after segmentation of the different tissues in the two types of images.

#### Segmentation of Tissues

**FIGURE 3:**
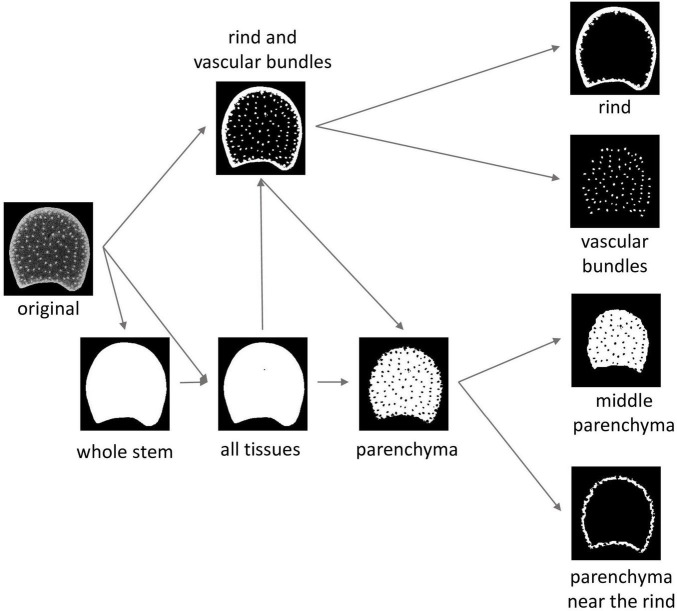
Image segmentation workflow and resulting tissue regions of interests (ROIs). The “whole stem” ROI corresponds to the mask of the section. In the “all tissues” ROI, the holes encountered in some of the sections are segmented. Parenchyma ROI and rind and vascular bundle ROI are temporary ROIs necessary to compute tissue ROIs. Middle parenchyma ROI and rind parenchyma ROI are subregions of the parenchyma ROI.

Regions of interest (ROIs) corresponding to each tissue were identified for the dark field and macrofluorescence images. A semiautomated workflow was adapted from Legland et al. (2014). The main steps summarised in Figure 3 were similar for the two kinds of images. The specific implementations for the two kinds of images are given in Supplementary Table 2.

For some stems, the parenchyma was torn during cutting, resulting in the presence of holes. The *whole stem ROI* was obtained by thresholding followed by hole filling to observe the whole stem area. A second region, called *all tissue ROI*, was considered, in which the possible holes were segmented by a second thresholding operation. The objective was to obtain an ROI that avoided possible holes to measure the parenchyma cell size and fluorescence properties.

From the *all tissue ROIs* and the original images, intermediate ROIs were created to correspond to the *rind and vascular bundle ROIs*. Alternating filtering based on morphological openings and closings (Soille, 2003) was applied to contrast rinds and vascular bundles from the parenchyma. It was followed by automatic thresholding. In the resulting ROIs, some vascular bundles could be connected and the rind could be split into several fragments. Rind and vascular bundles were differentiated by size analysis. The size of the vascular bundles was determined from the mode of the size distribution of the segmented objects in the BlueBox image located at a distance greater than 1 mm from the epidermis. It was used to define a size threshold to extract the *individualised vascular bundle ROIs*. *Rind ROIs* were built by merging external fragments larger than five times the value of the mode. In the BlueBox images, some vascular bundles were connected, and an additional region was computed to correspond to *all vascular bundle ROIs*, both connected and not connected.

The *parenchyma ROIs* were obtained as the logical difference between the *whole stem ROIs* and the *rind and vascular bundle ROIs*. The *parenchyma near the rind ROIs* and the *middle parenchyma ROIs* were obtained using *a priori* distances from the rind: below 500 μm for the parenchyma near the rind and over 1,000 μm for the middle parenchyma. Distances were chosen to contrast the two kinds of parenchymas.

#### Morphological Descriptors

##### Measuring the Raw Morphological Descriptors

Raw morphological descriptors could be directly measured from the segmented regions of interest as specified in Table 1. Areas were obtained by pixel counting; perimeters, vascular bundle length and width were obtained using the *regionprops* MATLAB function. Rind thickness was evaluated by granulometry using mathematical morphology transformations (Soille, 2003; Devaux et al., 2008; Legland et al., 2014, 2020); see “Parenchyma Cell Size” for an introduction to the method. The thickness distributions were obtained by applying opening transformations using squared structuring elements and a maximum size of 1,456 μm. The mean size of the distribution was taken as a measure of the average thickness of the rind.

**TABLE 1:** Measurement of the raw morphological descriptors: regions of interests (ROIs) used for measurement, morphological descriptors and acronyms.

Region of interest (ROI)	Morphological descriptors	Acronym
Whole stem ROI	Area Perimeter	St(A) St(P)
Rind ROI	Area Thickness	Ri(A) Ri(T)
All vascular bundles ROI	Area	Vb(A)
Individual vascular bundles ROI	Individual area Individual Elongation = width/length	Vi(A) Vi(E)

*Individual area or elongation means that measurements were performed for each segmented bundle.*

However, some defects related to section cutting and image segmentation were observed, such as missing pieces of rind or bundles not separated. Therefore, we developed an estimate of the morphological features as described below.

##### Estimating Rind Area

In sections where some rind pieces were missing, the rind area Ri(A) was estimated using the thickness Ri(T) and the perimeter of the whole stem St(P):


(2a) $$\tilde{\text{Ri}\left(\text{A}\right)}=\text{Ri}\left(\text{T}\right)\times \text{St}\left(\text{P}\right)$$

However, when examining undamaged sections, the estimated values were always higher than the measured values. The reason was an overestimation of the rind thickness due to vascular bundles that remained connected to the rind after segmentation. The average difference *diffEstMeas* between the estimated and measured rind areas was computed from the undamaged images and used as a correction factor to estimate the rind areas:


(2b) $$\tilde{\text{Ri}\left(\text{A}\right)}=\text{Ri}\left(\text{T}\right)\times \text{St}\left(\text{P}\right)-diffEstMeas$$

##### Estimating Stem Area

The stem area was equal to the measure of the area of the whole stem ROI when the rind was preserved, and it was estimated when the rind was fragmented:


(3) $$\tilde{\text{St}\left(\text{A}\right)}=\left\{\begin{array}{c} \text{St}\left(\text{A}\right):\text{rind}\text{preserved}\\ \text{St}\left(\text{A}\right)-\text{Ri}\left(\text{A}\right)+\tilde{\text{Ri}\left(\text{A}\right)}:\text{rind}\text{fragments} \end{array}\right\}$$

##### Computing the Relative Areas

Relative areas of measured parenchyma, estimated rind and measured vascular bundles were computed as percentages of the estimated whole stem area.

##### Average Morphology of Individual Vascular Bundles

Individual area and elongation measured for each vascular bundle were averaged to obtain one value per section: $\bar{\text{Vi}\left(\text{A}\right)}$ and $\bar{\text{Vi}\left(\text{E}\right)}$.

##### Density of the Number of Vascular Bundles

The number of vascular bundles (Vb(N)) was estimated as the total area of vascular bundles divided by the average area of vascular bundles:


(4) $$\text{Vb}\left(\text{N}\right)=\frac{\text{Vb}\left(\text{A}\right)}{\text{Vi}\left(\text{A}\right)}$$

The density of the number of vascular bundles (Vb(D)) was computed as the number Vb(N) divided by the whole stem area.

##### Parenchyma Cell Size

Cell size was measured from the BlueBox greyscale image on the two parenchyma regions. Grey-level granulometry developed using mathematical morphology (Soille, 2003) was applied without segmenting the cells as described in Devaux et al. (2008) and Legland et al. (2020). Grey-level granulometry consists of successively applying size transformations of the image through a mask of known geometry, called a *structuring element* (Soille, 2003). The size and shape of the structuring element are chosen according to the characteristics of the image. In the BlueBox images (Figure 1), cells appeared as isotropic dark objects, and closing transformations using squared structuring elements were retained. Closing can be compared to sieving dark objects in the image: dark objects smaller than the structuring element are removed while preserving the size of larger objects. When closings of increasing size are applied, the sum of grey levels, measured after each operation, increases. The increase depends on the quantity of objects removed. The result is a granulometric curve expressed as a percentage of grey-level variations according to the closing step.

In the present work, closing transformations between 18 and 207 μm were applied by steps of 7.26 μm. Compared to the procedure described in Devaux et al. (2009), grey-level granulometry curves were postprocessed by subtracting the residual size variations caused by the general background of the image in the region of interest and renormalisation of the curves. Grey-level mean sizes and standard deviations were computed from the granulometric curves as described in Devaux and Legland (2014).

#### Measure of Autofluorescence Pseudospectra

For each pixel, 11 fluorescence intensity values were measured. The set of fluorescence intensities measured for individual pixels or averaged over a set of pixels was called pseudospectra (Corcel et al., 2016). Because no photon can be emitted at wavelengths higher than the excitation wavelength, for the two visible filters blue and green, the channels BLb, GRb, and GRr showed no signals. They were nevertheless maintained in the pseudospectra and were considered a baseline.

Average autofluorescence pseudospectra were measured for the four tissue *ROIs*: *rind*, *all vascular bundles*, *parenchyma near the rind* and *middle parenchyma*. A preliminary analysis revealed a channel-dependent background intensity. Three regions without any signal were manually selected in four images of the series. The background pseudospectrum was computed as their average pseudospectra. It was subtracted from all other measured pseudospectra. A section-dependent overall intensity effect was observed, which was probably due to variations in section thickness. A normalisation procedure was set, which is detailed in the “Results” section.

#### Data Analysis

The morphological descriptors and the autofluorescence pseudospectra were analysed based on a principal component analysis and variance analysis, followed by multiple comparisons of the estimated marginal means. Analyses were performed within the MATLAB 2019b environment (Mathworks, Natick, MA, United States) using the statistics and machine learning toolbox.

Principal component analyses were applied separately to the morphological descriptors and the autofluorescence pseudospectra. The morphological descriptors were normalised to describe the variations independently of the units, and the loadings were represented as correlation circles. In the case of pseudospectra, the variables were not normalised to avoid assigning importance to the baselines of the pseudospectra, and the loadings were represented in the form of pseudospectra.

Variance analyses were applied to morphological descriptors and principal components to determine their significance with regard to the 14 inbred lines studied. Multiple comparisons of the estimated means were applied to reveal the most contrasted lines. In the case of autofluorescence pseudospectra, analyses of variance were applied to the principal component scores to determine the effects of inbred lines, tissues and their interactions.

## Results

### Variation in Cell Wall Phenolics and Digestibility Within the 14 Inbred Lines

The stems of the 14 lines were analysed for the cell wall content, lignin and hydroxycinnamic acid content of the cell walls, and digestibility. The cell wall content represented on average 54.9% of the stem dry matter, with a coefficient of variation (CV) of 8.5% (Table 2). The content of esterified para-coumaric acid showed the highest variability, with an average value of 1.60% of the cell wall dry matter and a coefficient of variation of 15.56%. Lower variability was observed for Klason lignin and esterified ferulic acid contents. On average, the lignin content was 18.3%, with a coefficient of variation of 9.2%, and the esterified ferulic acid content was 0.61%, with a coefficient of variation of 9.22%. Cell wall digestibility ranged from 25.4 to 43.9%, with an average value of 33.6% and a coefficient of variation of 14.9%.

**TABLE 2:** Mean values for stem cell wall contents, Klason lignin (KL), hydroxycinnamic acid **–** esterified p-coumaric acid (Ester pCA), esterified ferulic acid (Ester FA) **–** and cell wall digestibility (IVCW digestibility) for the 14 inbred lines.

Inbred line	Cell wall	KL	Ester pCA	Ester FA	IVCW
	% DM	% CW	%CW	% CW	digestibility
M01	52.7	20.51	1.59	0.62	32.7
		*0.11*	*0.03*	*0.01*	*0.85*
		(90.27)	(7.00)	(2.73)	
M02	56.8	20.65	1.61	0.55	34.7
		*0.56*	*0.01*	*0.00*	*0.05*
		(90.53)	(7.06)	(2.41)	
M03	55.5	18.24	1.68	0.72	37.8
		*0.09*	*0.00*	*0.01*	*0.10*
		(88.37)	(8.14)	(3.49)	
M04	57	20.13	1.06	0.57	29.9
		*0.14*	*0.01*	*0.00*	*0.94*
		(92.51)	(4.87)	(2.62)	
M05	52	15.88	1.29	0.64	41
		*0.07*	*0.01*	*0.00*	*0.29*
		(89.16)	(7.24)	(3.59)	
M06	50	15.51	1.33	0.67	43.9
		*0.34*	*0.00*	*0.01*	*0.01*
		(88.58)	(7.60)	(3.83)	
M07	62	16.58	1.81	0.58	39
		*0.05*	*0.02*	*0.00*	*0.94*
		(87.4)	(9.54)	(3.06)	
M08	53.7	17.92	1.56	0.59	32.8
		*0.09*	*0.08*	*0.03*	*1.91*
		(89.29)	(7.77)	(2.94)	
M09	63.6	17.1	1.38	0.64	32.7
		*0.38*	*0.03*	*0.01*	*0.17*
		(89.44)	(7.22)	(3.35)	
M10	53.6	16.93	1.65	0.61	30.7
		*0.32*	*0.02*	*0.03*	*0.76*
		(88.22)	(8.60)	(3.18)	
M11	47.6	20.4	1.94	0.57	27.6
		*0.27*	*0.09*	*0.07*	*1.38*
		(89.04)	(8.47)	(2.49)	
M12	49.9	19.26	1.68	0.5	30.5
		*0.32*	*0.03*	*0.01*	*0.57*
		(89.93)	(7.84)	(2.33)	
M13	61.8	19.15	1.89	0.66	25.4
		*0.16*	*0.11*	*0.04*	*0.12*
		(88.25)	(8.71)	(3.04)	
M14	52.3	17.94	1.91	0.68	31.1
		*0.28*	*0.06*	*0.03*	*0.91*
		(87.38)	(9.30)	(3.31)	

*% DM and % CW means that results are expressed in percent of dry matter and cell wall amount, respectively.*

*Numbers in italic correspond to the standard deviations. The cell wall % DM was measured in single (see “Materials and Methods” section). Number in brackets corresponds to the relative proportion of each cell wall phenolic compound expressed as percent of the sum of the cell wall phenolic compounds.*

The values of the biochemical traits measured in this study are in the range of those reported in the literature for inbred lines (Jung and Buxton, 1994; Méchin et al., 2000; Barrière et al., 2009a; El Hage et al., 2018). Despite the observed variability within the 14 inbred lines for the measured traits, no correlation between these traits was found. Inbred Lines M06 and M05 showed the lowest cell wall lignin content, while the highest values were found for M02, M01, and M11. M04 had a low content of para-coumaric acid but a high amount of lignin. In contrast, M11 had both high amounts of lignin and para-coumaric acid, and M14 had a high amount of para-coumaric acid and an intermediate amount of lignin. M03 and M05 had intermediate values for lignin and para-coumaric acid, while M03 and M05 the highest and lowest values of ferulic acid content, respectively. The highest cell wall digestibility was found for Lines M05 and M06, which had the lowest amount of lignin. Although M01 and M02 had the highest lignin content, they showed intermediate cell wall digestibility.

In summary, our panel of inbred lines showed variability in the stem cell wall contents and phenolic composition and a lack of correlation between these biochemical traits.

### Examples of Images From Details to the Collection

Four samples from inbred lines with contrasting morphologies were selected for a preliminary investigation of the dataset.

#### Zoom Images

**FIGURE 4:**
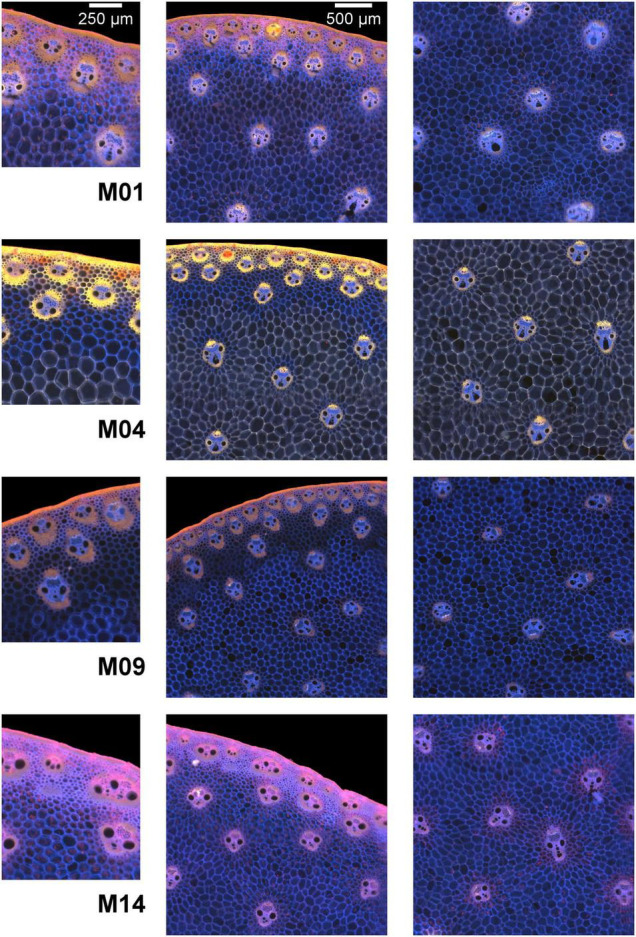
Composite macrofluorescence images. Example of inbred lines. Fields of view: 1.06 × 1.27 mm^2^ for the left column images and 3 × 3 mm^2^ for the middle and right column images. Blue fluorescence represents cell walls whose fluorescence is mainly induced by UV excitation, while pink or yellow fluorescence represents cell walls whose fluorescence is induced by visible excitation. Differences between the four inbred lines were observed for all tissues.

Zoom images were selected to compare the border and the middle of the sections from four contrasting samples (Figures 1, 4). At this scale, details in the rind, vascular bundles, and parenchyma cells are visible. Cell walls appeared in white in the morphological images and had colours ranging from pink to blue in the autofluorescence images. Based on the colour representation of the autofluorescence images, blue fluorescence represents cell walls with mainly UV-induced fluorescence while pink or yellow fluorescence represents cell walls with visible-induced fluorescence. Differences between the four inbred lines were observed for all the tissues.

The rind is composed of vascular bundles and small cells of cortical parenchyma (Esau, 1977). In the morphological images of Lines M04 and M09, the vascular bundles were clearly visible, while for M01 and M14, the rind formed a larger white ribbon and the cortical cells could hardly be distinguished. The autofluorescence images showed that for M01 and M14, the cortical cells contained fragments that fluoresced red, while for M04 and M09, the cells seemed empty. The red fluorophore probably corresponded to residual chlorophyll (Donaldson and Williams, 2018; Donaldson, 2020), which resulted in a seemingly wider rind observed using the BlueBox system. In the rind, the lignified sclerenchyma sheaths of vascular bundles (Lopez and Barclay, 2017; El Hage et al., 2018) were thick and fluoresced considerably, with the colour varying from yellow for M04 and orange for M01 and M09 to pink for M14.

In the pith, the vascular bundle sizes and shapes differed according to the line, with the vascular bundles from M04 and M09 smaller than those from M01 and M14. M01 vascular bundles were round, whereas, vascular bundles of the other three lines were more elongated. The fluorescence colour of vascular bundles was less strong but consistent with that observed in the rind. Inside vascular bundles, blue fluorescence was observed for the phloem and vascular parenchyma.

The parenchyma cells were clearly visible at this scale, and their size was dependent on the line and the region in the section. The smallest cells were observed for M09, and the largest were observed for M04. Cells near the rind seemed smaller than those in the middle parenchyma. Parenchyma cell walls fluoresced mainly in blue except for Line M04, which mainly showed yellow fluorescence. Specific fluorescence was observed in the parenchyma near the rind for M04 and M09. In the case of M09, the intensity was much lower, and in the case of M04, the fluorescence colour was blue compared to the yellow fluorescence of the middle parenchyma.

#### Large Image Scale

**FIGURE 5:**
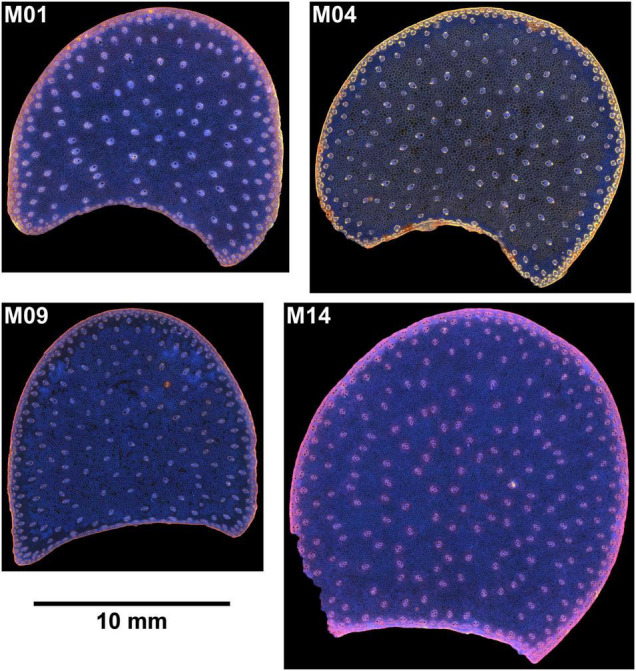
Composite macrofluorescence images: Example of large images for four inbred lines. The composite macrofluorescence images shows the homogeneity of fluorescence around the rind, vascular bundles, and within parenchyma.

Large images were created from individual fields of view of the images (Figures 2, 5). The concave regions in the sections correspond to the location of the main ear. The large image reveals the stem section area, rind, vascular bundle repartition and parenchyma. The section area was the largest for Line M14 and the smallest for M09. The images show that the rind thickness was homogeneous all around the section as well as the vascular bundle size. The composite macrofluorescence images (Figure 5) showed largely homogeneous fluorescence around the rind, vascular bundles and parenchyma. In particular, the specific fluorescence found for the parenchyma near the rind for Lines M04 and M09 could be observed all around the sections. For M01, yellow fluorescence occurred in small places of the rind.

### Extraction of Morphological Descriptors for the Four Examples of Inbred Lines

#### Proportions of Tissue Areas, Vascular Bundle Morphology, and Density

The proportion of tissues extracted for the four example lines are reported in Table 3: area of the stem section in cm^2^, parenchyma, rind and vascular bundle areas, which are expressed as a percentage of the area of the stem section, vascular bundle density, mean area and elongation of individual bundles.

**TABLE 3:** Morphological descriptors of the four examples of inbred lines.

Descriptor	M01	M04	M09	M14	Code
**Proportion of tissue and vascular bundle morphology and density**					
Stem area (cm^2^)	1.45 *0.06*	1.48 *0.07*	1.21 *0.02*	2.36 *0.03*	St(A)
Parenchyma area (%) of the stem area	71.9 *1.4*	81.7 *0.5*	80.3 *0.1*	71.5 *1.7*	Pa(A)
Rind area (%) of the stem area	21.2 *2.0*	13.3 *0.4*	13.0 *0.5*	20.2 *1.0*	Ri(A)
Vascular bundle area (%) of the stem area	8.2 *0.2*	6.2 *0.2*	6.9 *0.2*	8.7 *0.4*	Vb(A)
Vascular bundle Density (number per cm^2^)	63.4 *2.3*	81.7 *0.7*	116.6 *6.9*	62.1 *1.2*	Vb(D)
Vascular bundle Mean Area (mm^2^)	0.130 *0.007*	0.076 *0.002*	0.059 *0.002*	0.141 *0.009*	Vi(A)
Vascular bundle elongation	0.777 *0.003*	0.802 *0.009*	0.738 *0.026*	0.790 *0.015*	Vi(E)
**Parenchyma cell size**					
Middle parenchyma Grey level mean size (μm)	70.0 *0.2*	81.8 *0.7*	65.5 *0.8*	64.2 *0.6*	Pm(Cd)
Middle parenchyma Standard deviation (μm)	27.9 *0.6*	31.6 *0.2*	29.1 *0.9*	29.4 *0.6*	Pm(Cs)
Parenchyma near the rind Grey level mean size (μm)	57.9 *0.9*	54.9 *0.2*	46.0 *0.6*	56.2 *1.7*	Pr(Cd)
Parenchyma near the rind Standard deviation (μm)	31.6 *1.0*	30.6 *0.4*	27.4 *0.5*	34.9 *1.3*	Pr(Cs)
**Parenchyma cell wall density**					
Middle parenchyma Cell wall density (%)	4.29 *0.01*	3.67 *0.03*	4.58 *0.06*	4.67 *0.05*	Pm(CD)
Parenchyma near the rind Cell wall density (%)	5.18 *0.08*	5.46 *0.02*	6.53 *0.09*	5.36 *0.16*	Pr(CD)
**Tissue cell wall proportion**					
Total cell wall Area [% St(A)]	32.7 *2.0*	23.1 *0.5*	24.1 *0.3*	32.4 *1.3*	CW(T)
Middle parenchyma cell wall area [% CW(T)]	6.3 *0.6*	8.8 *0.3*	10.1 *0.1*	7.7 *0.5*	Pm(Cw)
Parenchyma near the rind cell wall area [% CW(T)]	3.9 *0.2*	6.3 *0.1*	7.4 *0.1*	3.1 *0.1*	Pr(Cw)
Rind cell wall area [% CW(T)]	65.3 *2.0*	58.6 *0.8*	56.4 *1.2*	63.2 *0.7*	Ri(Cw)
Vascular bundle cell wall area [% CW(T)]	25.3 *1.2*	27.0 *0.7*	28.6 *1.1*	26.8 *0.6*	Vb(Cw)

*Proportion of tissues as a percentage of the stem area, parenchyma cell size and cell wall density, and proportion of tissues as a percentage of the total cell wall. Mean values and standard errors measured for the two sections of the inbred lines. Numbers in italic correspond to the standard deviations.*

The stem area was two times larger for M14 than for M09 and similar for M01 and M04. The rind area was larger for M01 and M14, as expected from the images. Both lines also showed a larger vascular bundle total area together with a large area for individual vascular bundles. The smallest vascular bundles were observed for Line M09 along with the highest density, nevertheless resulting in a small relative total area. The proportion of parenchyma was consequently smaller for M01 and M14 and larger for M04 and M09. The vascular bundle shape did not vary much, as visually observed in the images.

#### Parenchyma Cell Size

**FIGURE 6:**
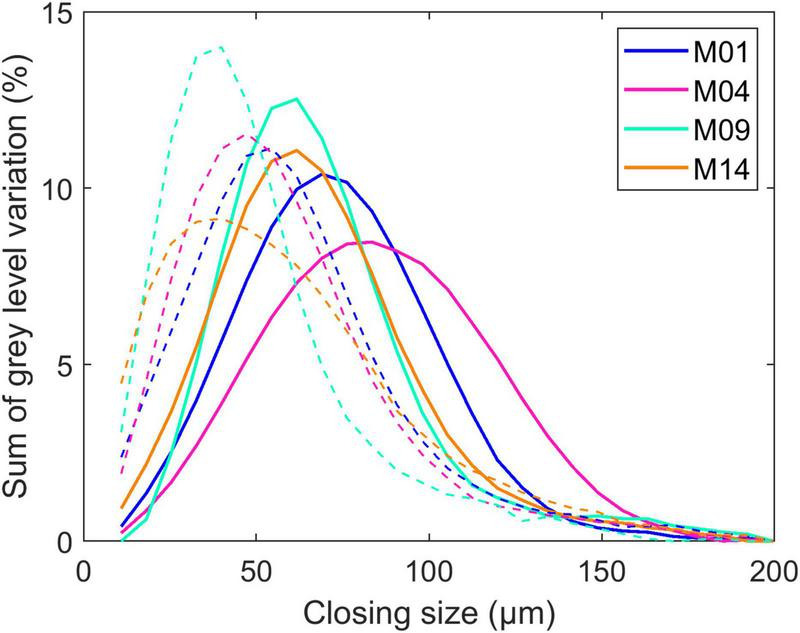
Parenchyma cell size. Example of granulometric curves computed for four inbred lines. The solid and dashed lines represent the granulometric curves computed in the middle parenchyma and the parenchyma near the rind, respectively. Granulometric curves can be compared to normal particle size distributions, with the position of the mode indicating the predominant cell size and the width reflecting the heterogeneity of cell sizes. Because the cells are isotropic, the closing size can be interpreted as the cell diameter.

Cell size was evaluated by grey-level granulometry without segmenting individual cells. The method was shown to be relevant to compare tissue sections from the BlueBox darkfield images (Devaux et al., 2008, 2009). Figure 6 shows the average granulometric curve computed for the parenchyma near the rind (dashed lines) and the middle parenchyma (solid lines). Granulometric curves can be compared to normal particle size distributions, with the position of the mode indicating the predominant cell size and the width reflecting the heterogeneity of cell sizes. Because the cells were isotropic, the closing size can be interpreted as the cell diameter.

In the middle parenchyma, the smallest cells were observed for Lines M09 and M14, with a cell diameter of approximately 60 μm, and the largest cells were observed for M04, with a cell diameter of approximately 85 μm, with M01 being intermediate. The distribution was more heterogeneous for M04, for which small cells were clearly distinguished around vascular bundles (Figure 1).

Cells were found to be smaller in the parenchyma near the rind, which was also measured in Legland et al. (2020), who computed local granulometric curves in a section of maize stem. M09 and M14 showed the smallest diameters of approximately 40 μm, and M01 showed the largest diameter of approximately 55 μm. M14 differed from the other lines by its greater heterogeneity in cell size. Figure 1 shows that the cell walls were not always clearly contrasted due to the presence of cell content that may result in measuring small size reflecting the distance between cell wall and cell content together with cell size.

To summarise the granulometric curves, grey-level mean sizes and standard deviations were computed (Devaux and Legland, 2014; Legland et al., 2014; Table 3). The grey-level mean sizes were approximately 65–80 μm for the middle parenchyma and 45–60 μm for the parenchyma near the rind. The standard deviations of the granulometric curves were approximately 30 μm and depended on the inbred line, i.e., larger values were observed for M04 in the middle parenchyma and for M14 in the parenchyma near the rind.

#### Estimating the Proportions of Tissue Cell Walls

##### From 2D Images to Volumes of Cell Walls: Principles and Hypotheses

Morphological features were extracted with the objective of examining their relationships with data such as chemical composition data or wall digestibility, which are measured on stems. In the present work, we proposed estimating the volume of tissues from 2D images considering several approximations and hypotheses. First, we considered that the internode under the ear was representative of the stem (Méchin et al., 1998). The internode was considered as a cylinder, and the density value of the cell walls was constant regardless of the cell type. This means that the volume and mass of cell walls are proportional. With these assumptions, the relative proportions of tissues in the internode can be estimated from their relative areas in the internode sections.

In the case of rinds and vascular bundles, the area proportions of tissue largely reflect the quantity of cell walls because these are thick and the lumen of the cells is only slightly visible. Therefore, we approximated that the area of these tissues that corresponded mainly to their cell wall proportion. In contrast, the amount of parenchyma cell wall depends on the cell size. This amount was estimated using the parenchyma tissue area and the parenchyma cell wall density that was evaluated from the cell size as described below.

##### Parenchyma Cell Wall Density

A parenchyma cell wall density estimate was derived from the grey-level mean sizes. Parenchyma cells were modelled as spheres with a radius (R) that corresponded to the grey-level mean size divided by 2. The cell wall density is equal to the ratio between the wall volume and cell volume, and the wall volume is equal to the cell surface multiplied by the wall thickness. In this case, the cell wall density Cw(D) expressed in percentage of volume is equal to the following:


(5) $$\text{Cw}\left(\text{D}\right)=\frac{3\times \text{CwThickness}}{\text{R}}*100$$

In the present work, the cell wall thickness CwThickness was set to 0.5 μm (Jung and Engels, 2001). Consistent with the cell size, the cell wall density was greater in the parenchyma near the rind (5–6%) than in the middle parenchyma (approximately 4%) (Table 3).

The total amount of cell wall in the parenchyma was assessed as the density of the cell wall multiplied by the parenchyma area, i.e., parenchyma cell wall areas, which are considered representative of their volume in the case of a cylindrical internode (Table 3). The middle parenchyma area was taken to estimate the cell wall amount. The area for the parenchyma near the rind was computed as the total parenchyma area minus the middle parenchyma area. It therefore also included the region between 500 and 1,000 μm.

##### Estimating Tissue Cell Wall Proportions

The total cell wall amounts were computed as the sum of the rind and vascular bundle areas plus the parenchyma cell wall amounts. Finally, the proportion of tissues was computed as the relative cell wall amounts. The values are reported in Table 3. Because of the different approximations, the rind and vascular bundle cell wall amounts could be somewhat overestimated. Nevertheless, these values were considered relevant to compare the lines. Table 3 shows that the rind was the major tissue, followed by the bundles. Depending on the cell size, stem diameter and parenchyma proportion, the contribution of parenchyma near the rind and middle parenchyma varied for the four inbred lines: the smallest contribution of the parenchyma near the rind was observed for M14 and the largest was observed for M09.

### Tissue Pseudospectra of the Four Examples of Inbred Lines

#### Normalisation of Pseudo Spectra

Tissue-specific fluorescence pseudospectra were studied for the rinds, vascular bundles, and parenchyma near the rind and in the middle of the section. In the parenchyma, the pseudospectra depended on the fluorescence properties of the cell walls but also on the density of the cell wall. Parenchyma pseudospectra were therefore divided by the cell wall density. In this way, we expected to estimate the fluorescence that would have been measured on the walls alone, thereby avoiding the cell lumens. In addition, the overall fluorescence intensity was found to be section-dependent regardless of the tissue, which was attributed to uncontrolled thickness variations. A section normalisation factor was assessed as follows. For each section and for each tissue pseudospectra (rind, vascular bundles, parenchyma near the rind and middle parenchyma after correction for the cell wall density), the mean fluorescence intensity measured for the 11 channels was computed: $\bar{\text{Ri}\left(\text{F}\right)}$, $\bar{\text{Vb}\left(\text{F}\right)},\bar{\text{Pr}\left(\text{F}\right)}$, and $\bar{\text{Rm}\left(\text{F}\right)}$. The normalisation factor of the section was computed as the mean fluorescence intensity:


(6) $$Fn\left(section\right)=\frac{\left(\bar{\text{Ri}\left(\text{F}\right)}+\bar{\text{Vb}\left(\text{F}\right)}+\bar{\text{Pr}\left(\text{F}\right)}+\bar{\text{Rm}\left(\text{F}\right)}\right)}{4}$$

Each tissue pseudospectrum was divided by this normalisation factor.

#### Spectral Information in the Pseudospectra

**FIGURE 7:**
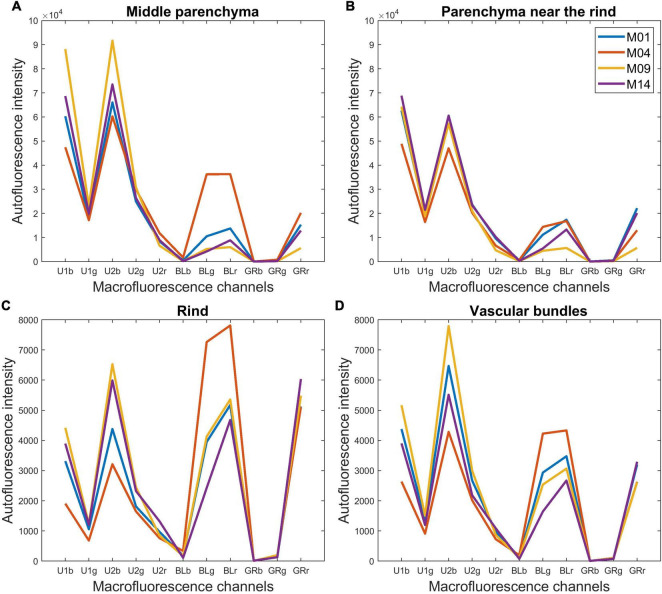
Macrofluorescence analysis. Tissue-normalised pseudospectra of the four examples of inbred lines: **(A)** middle parenchyma, **(B)** parenchyma near the rind, **(C)** rind and **(D)** vascular bundles. The pseudospectra represent the average spectral fluorescence behaviour computed over all pixels of the considered region of interest. The first five values report the intensity of UV-induced fluorescence, and the six others report the intensity of visible-induced fluorescence. The pseudospectra were normalised and only relative intensity variations can be discussed.

The resulting pseudospectra are shown in Figure 7 for the four example lines. In the pseudospectra, each value corresponds to the average fluorescence intensity of one of the 11 channels of the multispectral images. The colour images in Figures 4, 5 represent a summary of the 11 spectral fluorescence channels, while the pseudospectra represent the average spectral fluorescence behaviour computed over all pixels of the considered region of interest. The first five pseudospectra values report the intensity of UV-induced fluorescence, and the six others report the intensity of visible-induced fluorescence. As mentioned in the “Materials and Methods” section, no signal was observed in the channels BLb, GRb, and GRg, which were kept at baseline. Because the pseudospectra were normalised, only relative intensity variations can be discussed.

In the plant cell walls, not all the constituents are fluorescent for this range of excitation wavelengths. Polysaccharides are not fluorescent while lignin and hydroxycinnamic acids are the major natural fluorophores. To compare the normalised fluorescence intensities with the number of phenolic compounds (lignin + hydroxycinnamic acids), their relative amounts were calculated (Table 2). With this normalisation, Line M04 contained less para-coumaric acid than the three other lines, with a value of 4.87% compared to more than 7.00%, but more lignin, with a value of 92.51% compared to less than 90.3%. M14 was characterised by a high relative amount of para-coumaric acid (9.30%).

Hydroxycinnamic acids emit blue fluorescence with UV excitation at neutral pH, and lignin has a wide excitation range. Excitation with UV and blue light results in blue and green emission of lignins (Donaldson and Williams, 2018; Donaldson, 2020). Thus, a greater amount of visible fluorescence was assumed to correspond to samples that contained more lignin. Similarly, a greater amount of UV fluorescence was assumed to correspond to more hydroxycinnamic acids.

In addition, localisation or the lack of localisation of hydroxycinnamic acids and lignin could be responsible for the specific colour observed within a given line. Thus, the yellow colour of Line M04 observed in Figure 4 could be ascribed to a high relative amount of lignin together with a low relative amount of hydroxycinnamic acid. For M14, pink fluorescence could be ascribed to a low relative amount of lignin together with a high hydroxycinnamic acid content. In the following, tissue pseudospectra were examined to identify tissues that presented differences in phenolic compounds and differences between lines.

#### Tissue Pseudospectra of the Four Example Lines

For the parenchyma regions (Figures 7A,B), the UV-induced fluorescence was always stronger than the visible-induced fluorescence, showing that these tissues contained relatively less lignin and more hydroxycinnamic acids, which resulted in the generally blue-coloured parenchyma (Figures 4, 5). In contrast, rind and vascular bundles showed visible-induced fluorescence similar to UV-induced fluorescence (Figures 7C,D), which was consistent with the lignification of these tissues (Akin, 1989; Wilson et al., 1993; Hatfield et al., 1999; Zhang et al., 2013).

After normalisation, the fluorescence of the parenchyma was approximately 10 times more intense than that of the rind and vascular bundles. This ratio is somewhat overestimated because cell size was considered for the parenchyma cells and not for rind and vascular bundles. Nevertheless, the result is consistent with the fact that the parenchyma cell walls were clearly visible despite the wall thickness between two cells (1 μm) being much smaller than the pixel size (2.78 μm). Willemse and Emons (1991) also reported lower UV autofluorescence for sclerenchyma walls than for parenchyma walls, which was even more pronounced when related to the cell wall area. Another explanation for the relatively lower fluorescence intensity of lignified tissues is that lignin fluorescence is a complex process involving different fluorophores with different fluorescence profiles and energy transfer processes. Lignin fluorescence can be quenched by interactions with other polymers inside the cell walls, especially UV-induced fluorescence (Donaldson, 2020).

Comparing the rind and vascular bundles, visible-induced fluorescence was higher in the rind regions for the four lines. The composite macrofluorescence images in Figure 4 show that visible-induced fluorescence was mainly observed in the sclerenchyma sheath of vascular bundles and that the sheath was much thicker in the rind than in the pith. In addition, the relative proportion of blue parenchyma was higher in the bundle than in the rind.

In the case of the M09 parenchyma, pseudospectra allow the quantification of the lower intensity of the parenchyma near the rind compared to the middle parenchyma. In the case of M04, visible-induced fluorescence was found to be much lower in the parenchyma near the rind than in the middle parenchyma. This finding corresponds to the blue and yellow–white fluorescences observed in Figure 4 for the parenchyma near the rind and the middle parenchyma, respectively. For the other two lines, the pseudospectra of the two parenchyma were largely similar.

Looking more specifically at the lines, the intensity of the visible-induced fluorescence was much higher for M04 than for the other three lines in the rind, bundles and middle parenchyma. This finding is consistent with the high relative amount of lignin. It also suggests that a significant amount of lignin was found in the parenchyma cell walls for this line. The occurrence of an equal intensity after blue excitation in the green BLg and red BLr channels led to the strong yellow fluorescence of the rind and vascular bundles and to the yellow–white fluorescence of the cell walls in the middle parenchyma (Figure 4).

The pink fluorescence of the rind and vascular bundles observed for M14 was due to a lower relative green fluorescence after blue excitation (Blg channel), which corresponded to the lower relative lignin content. This was also measured for parenchyma cell walls. More generally, for a given line, the relative proportions of fluorescence measured after blue excitation in channels green BLg and red BLr were similar for all tissues, which suggests that the signature of blue-induced lignin fluorescence would not be tissue-dependent but line-dependent.

The highest UV-induced fluorescence intensity was observed for M09 and M14 in the rind and the two parenchymas. The two lines contained the most hydroxycinnamic acids. In the case of M09, almost no visible-induced fluorescence was observed in the parenchyma, suggesting that lignin was only found in the rind and vascular bundles.

In conclusion, the normalised pseudospectra were considered relevant to quantify the differences in the tissue fluorescence observed in the multispectral images.

### Histological Variability Within the 14 Inbred Line Collections

#### Morphological Analysis

##### Descriptors Extracted for the 14 Inbred Lines

Examples of images acquired for each of the 14 inbred lines can be found in the Supplementary Figure 1. All descriptors were computed for the two stems of the 14 inbred lines. Average values are reported in the Supplementary Table 3. The main points are reported here. The area of the stem section ranged from 1.21 to 2.36 cm^2^, with an average of 1.78 cm^2^. The parenchyma covered on average 76.1% of the total area of stem sections, and the coefficient of variation was 8% for the set of 14 inbred lines. Larger variations between inbred lines were observed for the rind and vascular bundle relative areas, with average values of 17.0 and 7.0%, respectively, and coefficients of variation of 27 and 20%, respectively. The vascular bundle density varied from 117 to 50 per cm^2^ with an average value of 77. Legland et al. (2017) also studied maize internodes under the ears of four inbred lines grown under two irrigation conditions and found values ranging from 1.0 and 3.0 cm^2^ for the area of the sections. The rind accounted for 10.3 to 16.8% of the section area, the vascular bundles accounted for 3.1 to 7.3% and the parenchyma accounted for 80–86%. The bundle density ranged between 111 and 66 per cm^2^. Vo et al. (2020) compared internodes under the ear of six maize inbred lines and reported values ranging from 1.6 and 4.0 cm^2^ for the area of the sections and 11–19 and 77–86% of the section area of the rind and pith parenchyma, respectively. The bundle density ranged between 76 and 42 per cm^2^. In the internode sections of sorghum, a species that is very close to maize, Wilson et al. (1993) found that the rind accounted for 16.2% of the total section area, the parenchyma accounted for 79.2% and the vascular bundles in the pith parenchyma accounted for 4.7%. The values found in our work are on the same order of magnitude of those reported in these manuscripts.

In the present work, we estimated the contribution of the different tissues to the total wall content of the internodes on the basis of the tissue surface proportion in the sections and from a simple internode model. The rind and vascular bundles represented 61 and 25% of the total cell wall on average, respectively, with coefficients of variation of 8 and 12%, respectively. The middle parenchyma and parenchyma near the rind represented 9 and 5%, respectively, of the total cell wall, with high coefficients of variation of 25 and 30%, respectively. The values reported here for the relative contribution of tissues to the total cell walls were in the range of those reported by Wilson et al. (1993) for sorghum internodes. In this study, the tissues of one cultivar were manually separated and analysed individually. The rind and vascular bundles accounted for 68.7 and 11.4% of the total cell wall, respectively. The pith parenchyma accounted for 22% of the total cell walls.

Considering all descriptors, the coefficient of variation ranged between 6% (standard deviation of cell diameters) and 36% (average area of individual vascular bundles). An ANOVA test was run individually on the descriptors to test their ability to discriminate lines. All descriptors were found to be significant for the line effect, with *p values* lower than 0.01.

##### Principal Component Analysis

A principal component analysis was performed on the subset of 13 morphological descriptors of the 14 inbred lines, including the stem area, proportion of cell walls in the stem, relative proportions of cell wall ascribed to tissues, parenchyma mean cell diameters and standard deviations, and vascular bundle density and morphology. A variance analysis was applied to the principal components. The four first principal components accounted for 44, 15, 14, and 10% of the total variance, and the line effect of these components was highly significant.

**FIGURE 8:**
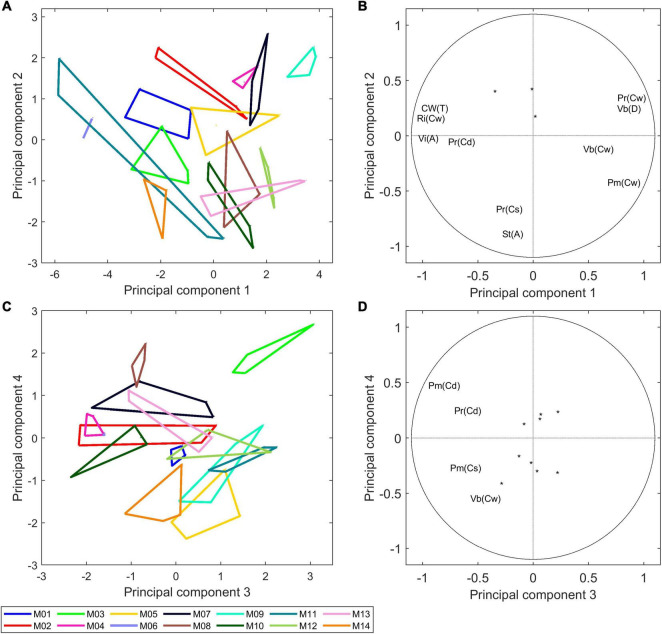
Morphological analysis of 14 inbred lines: principal component analysis. **(A,B)** Similarity map and loadings of components 1 and 2 (44 and 15% of the total variance). **(C,D)** Similarity map and loadings of components 3 and 4 (14 and 10% of the total variance). Convex hulls were drawn for each inbred line. Loadings are shown as correlation circles. Considering that the variables in the middle of the correlation circles are not representative of principal components, they were represented as points. Only variables with correlation over 0.5 with the principal component are shown. The similarity maps reveal a considerable variability between inbred lines based on their morphological descriptors.

Figure 8A shows the similarity map of components 1 and 2 and Figure 8C shows the similarity map of components 3 and 4 according to inbred lines. The corresponding loadings (Figures 8B,D) show the importance of the individual variables for the specified components.

Component 1 differentiates Lines M06, M11, M14, M01, and M03 based on the relatively high rind proportions [Ri(Cw)], high total cell wall amounts [Cw(T)] and large vascular bundle individual areas [Vi(A)], and it differentiates Lines M09, M07, M13, M12, and M04 based on the high vascular bundle densities [Vb(D)] and parenchyma cell wall amounts [Pm(Cw) and Pr(Cw)].

Component 2 differentiates lines based on their stem section area [St(A)], and it differentiated M09 and M07, which had a small section area, from M14 and M10, which had a larger section area. The two stems of M11 had very different stem diameters, with actual values of 2.6 and 1.5 cm^2^. For all other lines, the two stems were largely similar, as revealed by the convex hulls. Component 2 mainly described the stem area variations, with Lines M14, M10, M13, and M08 showing larger stem diameters than Lines M09, M07, M02, and M04.

Figure 8B highlights the correlation between the morphological descriptors. Namely, the expected strong contribution of the rind to the total cell wall amount in the stem as well as the negative correlation with the parenchyma cell wall amounts. A negative correlation *r* = −0.77 was observed between the vascular bundle density and the average individual area of vascular bundles. Indeed, a general trend was observed among the 14 inbred lines, with M06, M11, M14, and M01 having large bundles over 0.1 mm^2^ and less than 60 bundles per cm^2^ and M09, M07, M12, and M05 having small bundles smaller than 0.1 mm^2^ and more than 80 bundles per cm^2^. Zhang et al. (2020) measured the area of individual vascular bundles and their density in the stem for 480 inbred lines and reported a negative correlation between these two descriptors.

Beyond examining the components individually, it is interesting to note the distribution of the 14 inbred lines that reveals their specificity and the great variability of the collection. The same comment can be applied to the similarity maps of components 3 and 4. In this case, the components described variations in the parenchyma cell diameters Pm(Cd), Pm(Cs), Pr(Cd), and the proportion of vascular bundles Vb(CW). Line M03 was clearly highlighted mainly because of the much lower relative number of vascular bundles due to its low density in number. On this similarity map, the other lines contrasted were M08, M04, M02, M05, M14, M11, and M12.

We investigated the correlation between the morphological descriptors and the relative amounts of chemical compounds to further explore their tissue origin, but no correlation was found.

#### Autofluorescence Variations According to Tissue and Lines

The fluorescence colour quantified in the pseudospectra should reveal more lignin or hydroxycinnamic acids and their localisation in some specific tissues. To compare the 14 inbred lines of the study, multivariate analyses were performed on the tissue pseudospectra. Principal component analyses were performed to assess the relative importance of the tissue or line in determining the fluorescence properties. In a second step, the correlation between the relative amounts of phenolic compounds and the tissue pseudospectra was examined.

##### Principal Component Analysis of Tissue Pseudospectra

Because of the general intensity differences, principal component analyses were carried out separately on the rind and vascular bundle pseudospectra on the one hand and on the parenchyma pseudospectra on the other hand. A variance analysis was applied to the principal components to evaluate the effects of lines and tissues and their interaction.

**FIGURE 9:**
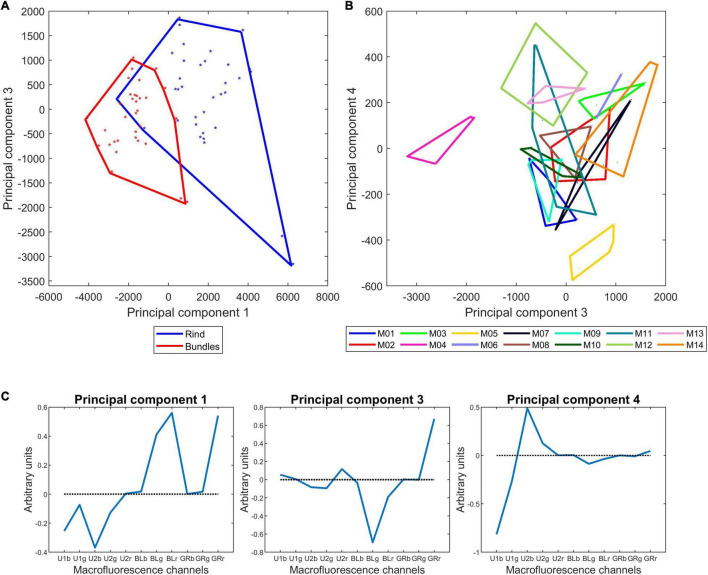
Macrofluorescence analysis of the rinds and vascular bundles of 14 inbred lines: principal component analysis. **(A,B)** Sample similarity maps of components 1–3 (72 and 11% of the total variance) according to tissues and 3 and 4 according to inbred lines (11 and 1% of the total variance). **(C)** Loadings 1, 3, and 4. The similarity map 1–3 reveals the strong variability between rind and vascular bundles. The significant variability between inbred lines based on their fluorescence properties is observed in the similarity map 3 and 4.

Table 4 reports the results of the variance analysis applied on the four first principal components computed for the rind and vascular bundles accounting for 72, 16, 11, and 1% of the total variance. For the four components, the line effect was significant. Rind and vascular bundles differed on components 1 and 3, and no interaction was revealed. Figure 9A shows the similarity map of components 1 and 3 according to the tissues. Figure 9B shows the similarity map of components 3 and 4 according to the lines. Figure 9C shows and the loadings of components 1, 3, and 4. Loading 1 revealed the relative variations between UV- and visible-induced fluorescence. Loading 3 was based on the relative variations observed in visible-induced fluorescence, e.g., green emission after blue excitation (BLg channel) versus red emission after green excitation (GRr channel). Loading 4 showed a difference in the relative blue emission using UV excitation of U1 and U2 (U1b and U2b channels). The similarity map of components 1 and 3 shows that for all lines, the rind and vascular bundles differed mainly by their relative visible and UV-induced fluorescence, and to a lesser extent by a relatively higher red fluorescence emission of the rind after green excitation. This difference could be ascribed either to cortical parenchyma cell walls or to their content. On this map, the line effect was mainly caused by M04, which corresponded to the extreme points for the two tissue scatterplots. The similarity map of components 3 and 4 reveals the line effect among the 14 lines. Despite some overlap, contrasting fluorescence fingerprints were observed for some lines, such as M04, M05, M14, and M12. M14, M03, and M06 showed relatively higher red fluorescence after green excitation (GRr channel) (see also Figures 7C,D for M14). Line M05 was characterised by its relatively high blue emission after U1 excitation compared to M12 or M13.

**TABLE 4:** Macrofluorescence analysis of 14 inbred lines.

Rind and vascular bundles				

	Component 1 72%	Component 2 16%	Component 3 11%	Component 4 1%
Line	12***	5***	19***	8***
Tissue	270***	–	17***	–
Interaction	–	–	–	–

**Parenchyma tissues**				

	**Component 1** **71%**	**Component 2** **18%**	**Component 3** **7%**	**Component 4** **3%**

Line	11***	9***	9***	15***
Tissue	70***	94***	22***	6*
Interaction	3**	9***	5***	4***

Variance analysis. Effects of inbred lines and tissues on the principal components. F value and significance. *^,^ **^,^ *** means that the probability was below 5%, 1% and 0.1%, respectively.

**FIGURE 10:**
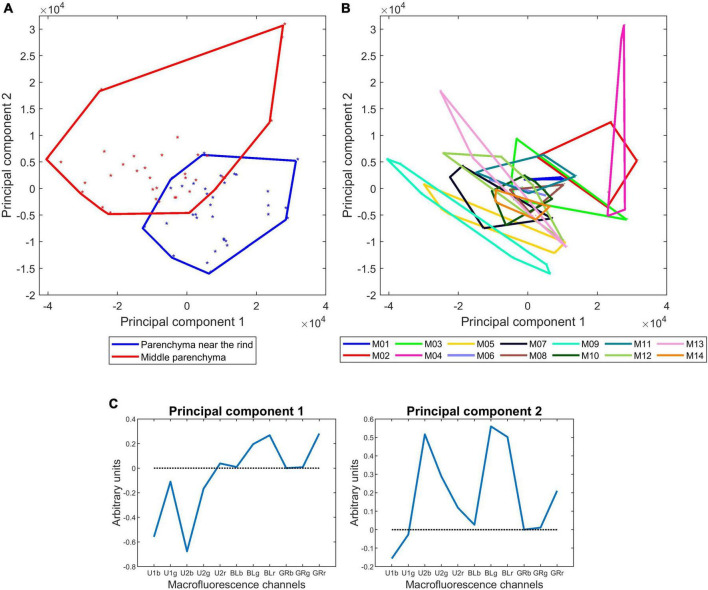
Macrofluorescence analysis of the parenchyma tissues of 14 inbred lines: principal component analysis. **(A,B)** Sample similarity maps of components 1–2 (71 and 18% of the total variance) according to tissues and inbred lines. **(C)** Loadings 1 and 2. The similarity maps reveal the significant variabilities between inbred lines and between parenchyma near the rind and middle parenchyma.

Table 4 and Figure 10 show the results obtained for the two parenchyma tissues. The first four components accounting for 71, 18, 7, and 3% of the total variance were found to be significant for both the lines, tissue and their interaction. Similarity maps and loadings are shown for components 1 and 2 (Figures 10A,B). The same map was drawn twice by considering the tissues or the lines. Figures for components 3 and 4 are given in Supplementary Figure 2. Loading 1 (Figure 10C) was partly similar to the one obtained for the rind and vascular bundles, thus showing the relative response after UV and visible excitation. Component 1 mainly described the differences between lines, with M04, M02, and M03 showing stronger visible-induced fluorescence and M09 and M05 showing stronger UV-induced fluorescence, especially in the middle parenchyma. Loading 2 (Figure 10C) described the relative intensity of the two parenchyma tissues, thus attesting to the generally higher fluorescence in the middle parenchyma except for the U1-induced fluorescence, which was slightly higher in the parenchyma near the rind. The interaction was also significant, highlighting that the differences near the rind and middle parenchyma were enhanced for some lines, such as M09 and M05. As expected, M04 was found to be different from the other lines. Other lines, such as M05, were found to be characteristic. For this line, the visible fluorescence in the middle parenchyma was very low, which could be related to the low lignin content of this line. In addition, the intensity difference between the middle and near the rind parenchyma was important, the cell content was found near the rind, and relatively high U1-induced fluorescence compared to U2-induced fluorescence could be observed, especially near the rind.

In summary, this analysis revealed that fluorescence properties were primarily tissue-dependent but also clearly line-dependent. In particular, the relative fluorescence emission after blue excitation seemed to be similar within an inbred line regardless of the tissue. These differences should be related to variations in the lignin content, composition and structure or in the phenolic acid contents.

##### Exploring the Correlation Between Biochemical Data and Tissue Fluorescence

Because the fluorescence properties of compounds depend on several factors, the interpretation of fluorescence variations is not straightforward. We investigated the correlation between tissue fluorescence pseudospectra and the relative amounts of fluorescent chemical compounds to further explore their tissue origin. The tissue fluorescence pseudospectra were normalised according to the individual sections, and this normalisation still allowed for the preservation of the relative intensity variations among the rind, vascular bundles and parenchyma. In parallel, the relative lignin, ferulic and para-coumaric acid amounts were considered after normalisation to their total amount (Table 2). A drawback is that this normalisation generates correlations between the variables. Thus, the correlation coefficient values were −0.94 between lignin and para-coumaric acid amounts, −0.54 between lignin and ferulic acid amounts, and 0.23 between para-coumaric and ferulic acid amounts.

**FIGURE 11:**
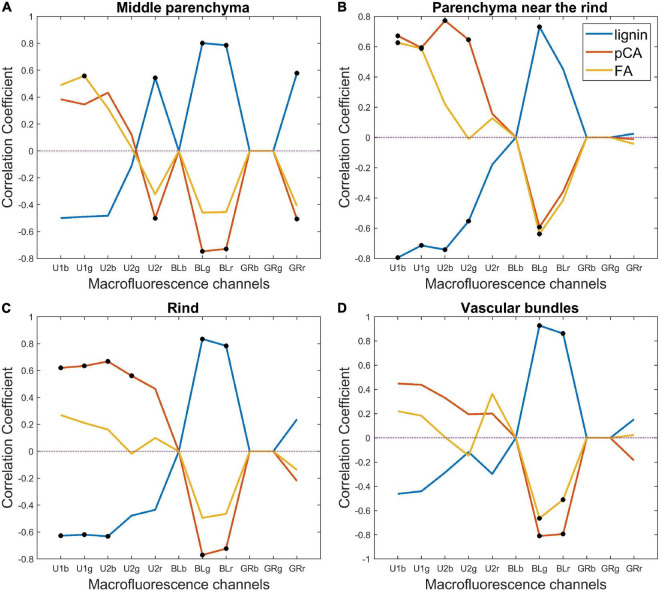
Macrofluorescence analysis. Correlation among lignin, para-coumaric acid (pCA), ferulic acid (FA) and tissue autofluorescence pseudospectra: **(A)** middle parenchyma, **(B)** parenchyma near the rind, **(C)** rind, and **(D)** vascular bundles. Significant coefficients are highlighted by black points. For all tissues, a significant positive correlation was found between lignin and blue-induced fluorescence. For para-coumaric acid, significant positive correlations were found for UV-induced fluorescence in the rind and in the parenchyma near the rind but not in vascular bundles and middle parenchyma.

Correlation coefficients were drawn according to the channels of the tissue pseudospectra (Figure 11). Significant coefficients are highlighted by black points. The correlation was always reversed for lignin and hydroxycinnamic acids, which is expected from the correlation induced by normalisation. For all tissues, a significant positive correlation was found between lignin and blue-induced fluorescence. This finding suggests that lignin is observed regardless of the tissue and that a high relative amount measured for the whole stem occurs in all tissues. In the case of the middle parenchyma, the lignin correlation is also related to red emission after U2 and green excitation. For para-coumaric acid, significant positive correlations were found for UV-induced fluorescence in the rind and in the parenchyma near the rind but not in vascular bundles and middle parenchyma. A significant positive correlation was found for ferulic acid in the parenchyma near the rind and to a lesser extent in the middle parenchyma. These results indicate that para-coumaric acids are mainly localised in the rind and parenchyma near the rind, and localisations of ferulic acids are mainly located in the parenchyma near the rind and in the middle parenchyma. In addition, the ferulic acid fluorescence signal was revealed mainly after U1 excitation, i.e., for shorter excitation wavelengths, while para-coumaric acid was revealed using both U1 and U2 filters.

## Discussion and Conclusion

The objective of the present work was the histological quantification of the morphology and fluorescence signature of maize forage stem sections for a set of 14 inbred lines used as parents for maize hybrid production. In addition to the relationship with end-use properties such as digestibility, this work aimed to explore the methodological potential of two techniques, namely, darkfield and fluorescence imaging, to study maize stem collection. Macrovision was retained to acquire images of whole stem sections with a fair pixel resolution, thus allowing for the quantification of cell size together with tissue proportions. The INRAE BlueBox prototype is dedicated to morphological plant tissue imaging with good contrast without any labelling because of darkfield illumination. This kind of illumination, which is less sensitive to variations in the density of walls than the usual transmitted light, is more suited to cell size analysis directly from grey-level images without any cell segmentation. Automated fluorescence macroscopes for multispectral image acquisition are the tools of choice for studying the autofluorescence properties of phenolic compounds in plant tissues. Both techniques should be considered medium-throughput methods that allow for the acquisition of 1 cm^2^ large images in approximately 5 and 20 mins per image for the BlueBox and the fluorescence macroscope, respectively. In the present work, sections were observed after being stored in ethanol, i.e., ethanol soluble material was removed, which led to the emptying of the cells except in the rind or in the parenchyma near the rind, where residual cell contents remained for some inbred lines. The fluorescence properties of this material suggest that it could be residual chlorophyll.

Image analysis was implemented to extract morphological features and autofluorescence pseudospectra. Four tissues were studied: rind, vascular bundles, parenchyma near the rind and middle parenchyma. In the present work, serial sections were used for the two devices, and images were segmented separately. One improvement would be to acquire images exactly for the same section and develop a segmentation workflow that takes into account both fluorescence and darkfield properties. For both images, the segmentation workflow was based on the same image processing steps, i.e., grey-level and size thresholds and alternating filtering, and it was fully automated. The only user intervention was to validate and adapt if necessary the automatic or preset thresholds. The rind, vascular bundles and parenchyma regions of interest were extracted. The rind segmented from the darkfield images corresponded to a material-dense region, with small cells having thick walls and bundles with thick sclerenchyma sheaths. The segmentation was also partially dependent on the occurrence of cell contents in and near the rind. The parenchyma near the rind was not segmented in the same manner. The 500 μm below the rind was examined to demonstrate the presence or absence of differences with the middle parenchyma. For this parenchyma, the cell size was smaller, and the fluorescence differences with the middle parenchyma were dependent on the line.

In this manuscript, 3D descriptors were estimated from 2D images given some assumptions. Indeed, the morphological descriptors were computed based on an estimation that considered the cell wall density of parenchyma tissues and the stem as a cylinder and the same histology of cross-sections all along the cylinder. The total derived cell wall amount can be interpreted as the relative areas of rind, bundles and parenchyma cell walls in the stem section. The fluorescence pseudospectra were also normalised by considering the parenchyma cell wall density.

We found a strong inbred line effect on the morphological descriptors. The most discriminant features were (1) the relative amount of rind and parenchyma tissues together with the density and size of individual bundles, (2) the stem area, and (3) the middle parenchyma cell diameter and distribution of the total vascular bundle amount. No correlation was observed between cell size and stem section, indicating that the diameter of the stem would rather depend on the number of cells in the parenchyma. A significant inverse correlation was observed between the vascular bundle size and density. Heckwolf et al. (2015) and Zhang et al. (2021) also found variations in stem diameter as well as in the area of the rind and pith of the inbred lines they analysed. Zhang et al. (2021) further analysed the variation in vascular bundle traits and reported wide phenotypic variations in vascular bundle size, number, and distribution density. Thirty of the phenotypic traits related to bundles showed high heritability, suggesting that the observed variations were at least partly of genetic origin. At the scale of whole cross-sections, they observed a negative correlation between the vascular bundle area and density, which was also observed in this study. However, neither of these studies considered the size of the cells in the parenchyma.

We did not find any correlation between the morphological descriptors and the phenolic composition of the 14 inbred lines, which could be explained by several hypotheses. First, biochemical measures were obtained from the whole stem, including the node, and we analysed sections taken in the middle of the internode. Second, none of the three phenolic constituents, i.e., lignin, para-coumaric or ferulic acids, can be considered tissue-specific biochemical markers. This conclusion is consistent with the results of the fluorescence pseudospectral analysis, at least for lignin. Indeed, the correlation profiles of the relative amount of lignin with the tissue pseudospectra clearly showed that a higher level of lignin resulted in a higher visible-induced fluorescence in all tissues.

Specific fluorescence signatures with a predominant tissue effect have been identified, and the inbred line effect was also always found to be significant. The rind, as the most lignified tissue, showed strong visible-induced fluorescence. Our results suggest that the colour of the visible-induced fluorescence, which was line-dependent, may depend on the amount of colocalised lignin and para-coumaric acid.

The relative amount of para-coumaric acid was found to be significantly correlated with the UV-induced fluorescence intensity in the rind and in the parenchyma near the rind, while ferulic acid was significantly correlated mainly with the parenchyma near the rind. In grasses, para-coumaric acid is ester linked to lignin and, to a lesser extent, to hemicelluloses (Hatfield et al., 2017). Since the rind is highly lignified, the presence of para-coumaric acid was expected. More surprisingly, para-coumaric acid was present in the parenchyma near the rind.

The parenchyma near the rind was less fluorescent on average than the middle parenchyma, although the extent of the difference was dependent on the inbred lines. Fasga staining is performed to reveal tissue lignification and often reveals this parenchyma region (El Hage et al., 2018). In this manuscript, the parenchyma Fasga that was coloured in red was correlated to the lignin amount, cell wall digestibility, and para-coumaric acid content (to a lower extent). They did not find any correlation between the lignin content and the red intensity in the rind or the number or density of bundles in the stem.

To further interpret the differences in autofluorescence between the tissues, it would be useful to have additional information about the biochemical composition of the different tissues. For example, the amount of etherified ferulic acid was not determined. Fasga or other lignin selective staining, such as Wiesner or Maüle staining, could be advantageously used to confirm the localisation of lignified tissues and reveal chemical differences in the lignin type (Méchin et al., 2005). Immunolabelling using antibodies would allow further identification and localisation of hydroxycinnamic acids (Philippe et al., 2007; Tranquet et al., 2009). Microspectroscopic techniques, such as Raman or infrared imaging (Gierlinger, 2018), would allow further localisation of phenolic compounds together with cell wall polysaccharides.

## Data Availability Statement

The datasets presented in this article are not readily available because research partly funded by private company. Requests to access the datasets should be directed to M-FD, marie-francoise.devaux@inrae.fr.

## Author Contributions

MB, M-FD, BD, and FG planned and designed the research. MB performed all sample preparation and image acquisition. M-FD and MB performed the image analyses and chemometric analyses. M-FD wrote the computer code. DL helped for image segmentation. M-FD, MB, and FG interpreted the results and wrote the manuscript. CB, DL, and BD did a thorough review of the manuscript. All authors contributed to the article and approved the submitted version.

## Conflict of Interest

The authors declare that the research was conducted in the absence of any commercial or financial relationships that could be construed as a potential conflict of interest.

## Publisher’s Note

All claims expressed in this article are solely those of the authors and do not necessarily represent those of their affiliated organizations, or those of the publisher, the editors and the reviewers. Any product that may be evaluated in this article, or claim that may be made by its manufacturer, is not guaranteed or endorsed by the publisher.
