# Supplementary material for: Darkfield and Fluorescence Macrovision of a Series of Large Images to Assess Anatomical and Chemical Tissue Variability in Whole Cross-Sections of Maize Stems
Source: Front Plant Sci. 2021 Dec 14;12:792981. doi: 10.3389/fpls.2021.792981 (PMC8712689; doi:10.3389/fpls.2021.792981)
Supplement: Supplementary file 1 [file Image_1.pdf]

Supplementary Images 1. BlueBox and composite macrofluorescence images. Example of large images for the 14 inbred lines.

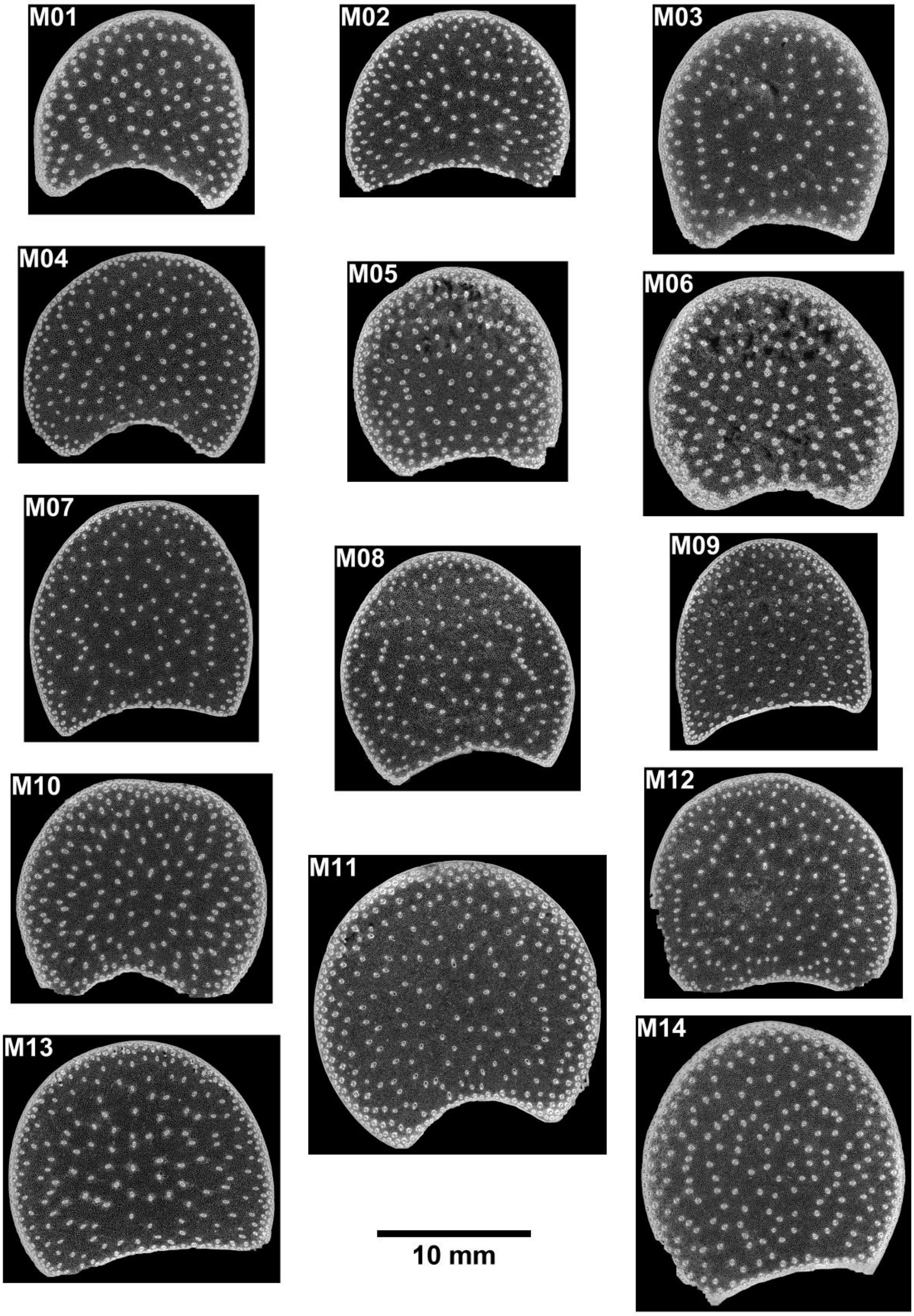

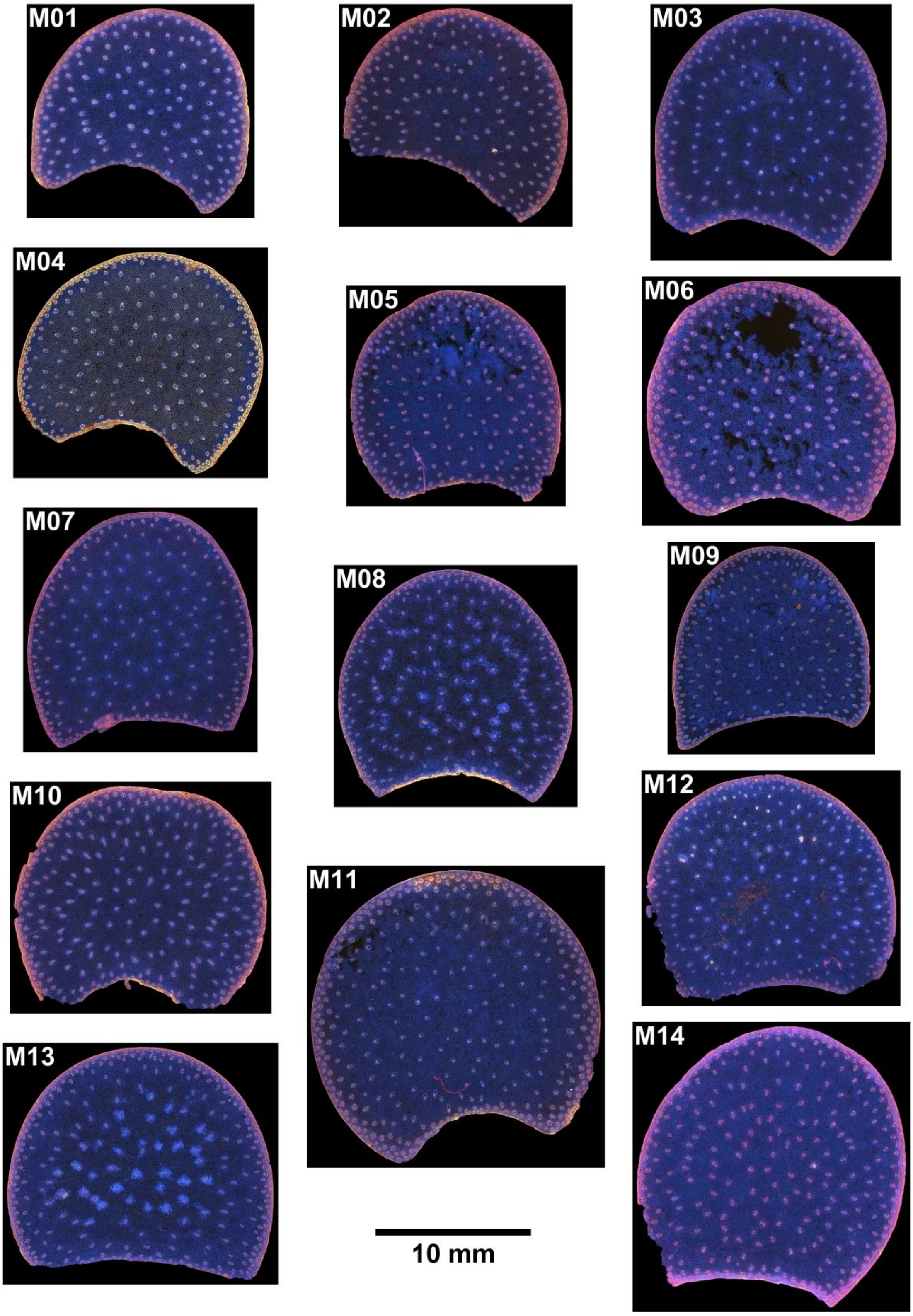

One image per line is represented. Images were reduced to perform visual comparisons among the 14 genotypes. At this scale, the general shape of the stem cross-sections, rind and vascular bundles can be observed, and parenchyma cells cannot be distinguished. M11 and M14 showed the largest cross-sectional areas, whereas M09 showed the smallest cross-sectional areas. M06 showed the thickest rind, and M09 showed the narrowest. The vascular

bundle size varied among the 14 genotypes. For example, M01 and M11 showed large and small bundles, respectively.

A range of autofluorescence behaviours was observed among the 14 lines. The fluorescence of the rind and vascular bundles was mainly pink/orange. M04 was the only genotype to show homogeneous yellow fluorescence for the rind. A few yellow regions are shown in the images of genotypes M01 and M11 and in the concave region for the image of M08. The blue fluorescence of the parenchyma differed according to the genotypes, as shown in the M11 and M14 sections. For M02 and M08, the parenchyma fluorescence appeared low compared to the other genotypes. It must be mentioned that this overall fluorescence visualisation depends on the parenchyma cell size and the section thickness. Specific fluorescence around vascular bundles was observed in at least M13 and M08 images.
