# Supplementary material for: Darkfield and Fluorescence Macrovision of a Series of Large Images to Assess Anatomical and Chemical Tissue Variability in Whole Cross-Sections of Maize Stems
Source: Front Plant Sci. 2021 Dec 14;12:792981. doi: 10.3389/fpls.2021.792981 (PMC8712689; doi:10.3389/fpls.2021.792981)

Supplementary Image 2: Macrofluorescence analysis of the parenchyma tissues of the 14 genotypes: principal component analysis. (A) and (B): Sample similarity maps of components 3-4 according to the tissue and genotype. (C): Loadings 3 and 4.

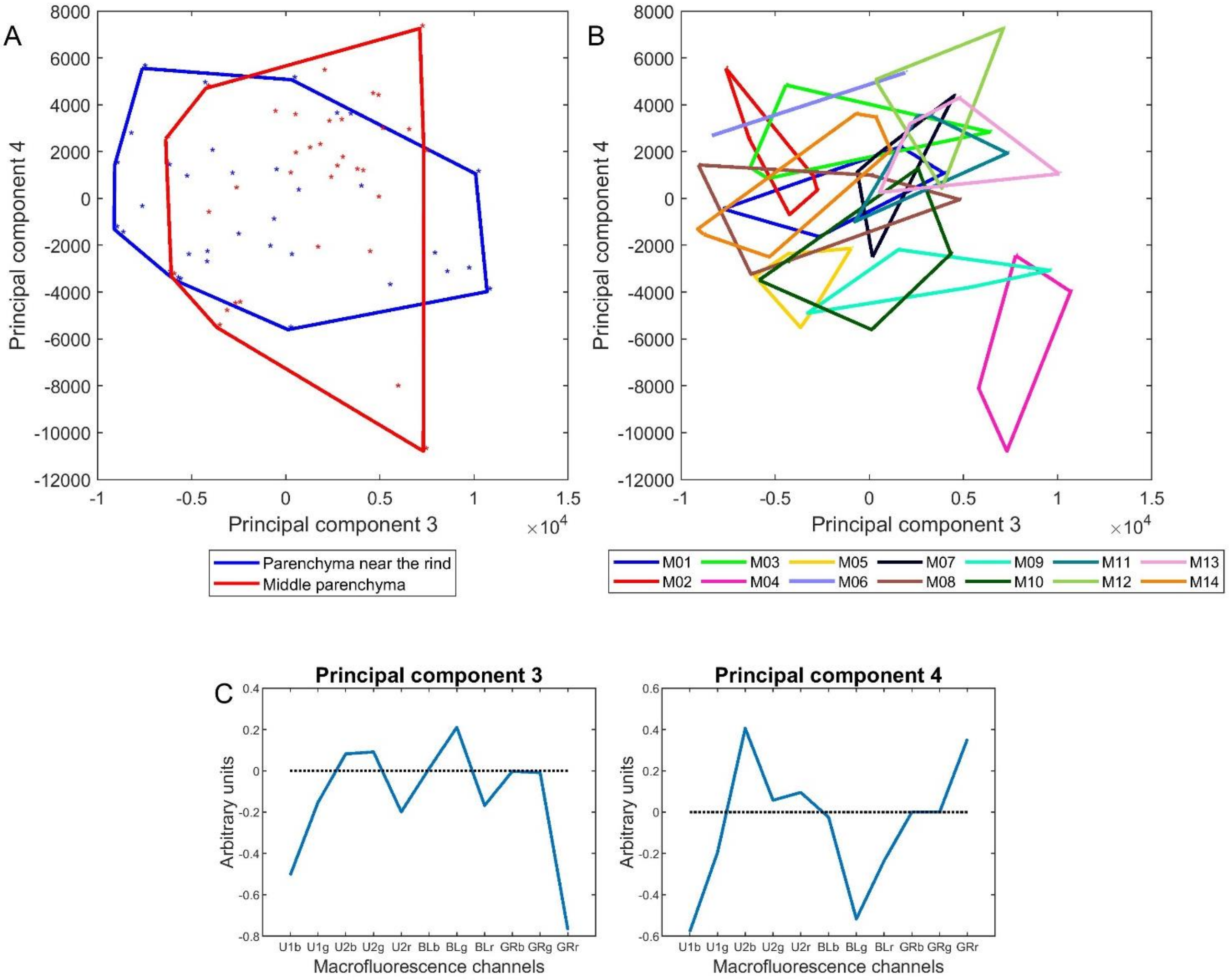

Supplement: Supplementary file 2 [file Image_2.pdf]
