# Supplementary material for: Darkfield and Fluorescence Macrovision of a Series of Large Images to Assess Anatomical and Chemical Tissue Variability in Whole Cross-Sections of Maize Stems
Source: Front Plant Sci. 2021 Dec 14;12:792981. doi: 10.3389/fpls.2021.792981 (PMC8712689; doi:10.3389/fpls.2021.792981)
Supplement: Supplementary file 3 [file Table_1.DOCX]

**Supplementary Table 1.** Characteristics of the four fluorescence filter cubes, acquisition time and multiplicative factor applied after acquisition

| Filter code | Excitation filter Bandpass (nm) | Dichroic mirror (nm) | Emission filter longpass (nm) | Acquisition time (ms) | Multiplicative factor |
| --- | --- | --- | --- | --- | --- |
| U1 | 327-353 | > 380 | > 364 | 1500 | 1 |
| U2 | 325-375 | > 400 | > 420 | 500 | 1 |
| BL | 460-490 | > 500 | > 515 | 500 | 2 |
| GR | 510-560 | > 565 | > 590 | 750 | 2 |
