# Supplementary material for: Darkfield and Fluorescence Macrovision of a Series of Large Images to Assess Anatomical and Chemical Tissue Variability in Whole Cross-Sections of Maize Stems
Source: Front Plant Sci. 2021 Dec 14;12:792981. doi: 10.3389/fpls.2021.792981 (PMC8712689; doi:10.3389/fpls.2021.792981)
Supplement: Supplementary file 4 [file Table_2.docx]

**Supplementary Table 2.** Segmentation workflow of the tissue regions of interest

| step | process | **BlueBox** | | | | | **Macrofluorescence** | |
| --- | --- | --- | --- | --- | --- | --- | --- | --- |
|  |  | **image** | **implementation** | | | | **image** | **implementation** |
| Whole stem ROI | thresholding | Original image  After filling holes | | Threshold 50  + user checking | | | Sum of intensity of the 11 channels  After filling holes | Otsu threshold (Otsu, 1979)  + user checking |
|  | Post filtering | Thresholded image | | Opening: size 123  Closing: size 83 | | | Thresholded image | Opening and closing of size 50 |
| **All tissue ROI** | Thresholding | Original image  without surrounding background | | Threshold 100  + user checking | | | Sum of the intensity of the 11 channels  without surrounding background | Threshold 25  + user checking |
| **Rind and vascular bundle ROI** | Alternating filtering using disks of increasing size  +  thresholding | Original image and all tissue ROI | | (Closing followed by openings) x 2  Disk radius of 2, 5, 11 and 13  Otsu threshold | | | Visible fluorescence images sum of intensity:  channels  BLg+ BLr+GRr | Closing + opening+closing  Disk radius of 2, 7 and 30  Otsu threshold in the middle of the section = at a distance of 1 mm from the border |
|  | Post treatment | Thresholded image | | Closing and opening: size 3  Area opening and closing:  Size 2000 pixels | | | - | - |
| **Individualised vascular bundles ROI** | Size analysis | Rind and vascular bundle ROI | | Find ***mode*** of the size distribution of segmented object.  Size thresholding to identify vascular bundles = >0.25X ***mode*** and < 2.25 x ***mode*** | | | Rind and vascular bundle ROI | Size thresholding to identify vascular bundles = >0.25X ***mode*** and < 3 x ***mode*** + user checking |
| **Rind ROI** | Size analysis | Rind and vascular bundle ROI | | | Rind = edge fragments of size > 5 X ***mode*** | | Rind and vascular bundle ROI | Rind = edge fragments of size > 5 X ***mode*** |
| **All vascular bundles ROI** | Logical operation | Rind and vascular bundle ROI | | | = Rind and vascular bundle ROI - Rind ROI | |  | |
| **Parenchyma ROI** | Logical operation | Whole stem ROI - Rind and vascular bundle ROI | | | | | Whole stem ROI - Rind and vascular bundle ROI | |
| **Near the rind parenchyma ROI**  **< 500 µm** | Erosion  + logical operation | Parenchyma ROI | | | | Erosion: size 500 µm  = Parenchyma ROI – eroded Parenchyma ROI | Parenchyma ROI | Erosion: size 500 µm  = Parenchyma ROI – eroded Parenchyma ROI |
| **middle parenchyma ROI**  **> 1000 µm** | Erosion  + logical operation | Parenchyma ROI | | | | Erosion: size 1000 µm  = eroded parenchyma ROI | Parenchyma ROI | Erosion: size 1000 µm  = eroded parenchyma ROI |

Workflow parameters of image processing to extract the tissue region of interest. For the threshold, user checking means that the user could manually modify the threshold if necessary. Morphological erosions, openings and closings were applied using squared structuring elements (Soille, 2003).

Otsu, N. (1979). A threshold selection method from gray-level histograms. *IEEE transactions on systems, man, and cybernetics* 9(1)**,** 62-66.

Soille, P. (2003). *Morphological Image Analysis: Principles and Applications.* Springer-Verlag.
