# Supplementary material for: Darkfield and Fluorescence Macrovision of a Series of Large Images to Assess Anatomical and Chemical Tissue Variability in Whole Cross-Sections of Maize Stems
Source: Front Plant Sci. 2021 Dec 14;12:792981. doi: 10.3389/fpls.2021.792981 (PMC8712689; doi:10.3389/fpls.2021.792981)
Supplement: Supplementary file 6 [file Table_4.docx]

**Supplementary Tables 4.** Morphological descriptors of the 14 inbred lines. (**A**) Tissue proportions and vascular bundle morphology and density (**B**) Parenchyma cell size (**C**) Parenchyma cell wall density (**D**) Tissue cell wall proportion

| 1. Tissue proportions and vascular bundle morphology and density | | | | | | | |
| --- | --- | --- | --- | --- | --- | --- | --- |
|  | Stem area (cm²) | Parenchyma Area (%) of the stem area | Rind area (%) of the stem area | Vascular bundle area (%) of the stem area | Vascular bundle density (number per cm²) | Vascular bundle mean area (mm²) | Vascular bundle elongation |
| Abbreviation | St(A) | Pa(A) | Ri(A) | Vb(A) | Vb(D) | Vi(A) | Vi(E) |
| **M01** | **1.45** | **71.9** | **21.2** | **8.21** | **63.4** | **0.130** | **0.78** |
| Standard deviation | 0.06 | 1.4 | 2.0 | 0.19 | 2.3 | 0.007 | 0.00 |
| M02 | 1.39 | 73.5 | 19.1 | 7.92 | 79.3 | 0.102 | 0.75 |
| Standard deviation | 0.01 | 1.7 | 1.7 | 0.34 | 5.4 | 0.010 | 0.02 |
| M03 | 2.01 | 76.7 | 19.9 | 5.36 | 51.3 | 0.105 | 0.73 |
| Standard deviation | 0.10 | 1.4 | 1.3 | 0.18 | 1.4 | 0.002 | 0.01 |
| **M04** | **1.48** | **81.7** | **13.3** | **6.22** | **81.7** | **0.076** | **0.80** |
| Standard deviation | 0.07 | 0.5 | 0.4 | 0.19 | 0.7 | 0.002 | 0.01 |
| M05 | 1.45 | 73.7 | 18.8 | 8.92 | 96.7 | 0.093 | 0.78 |
| Standard deviation | 0.02 | 1.6 | 1.9 | 0.27 | 4.6 | 0.005 | 0.00 |
| M06 | 1.99 | 63.7 | 26.3 | 8.82 | 50.0 | 0.177 | 0.74 |
| Standard deviation | 0.01 | 0.1 | 0.6 | 0.17 | 0.4 | 0.002 | 0.01 |
| M07 | 1.34 | 83.0 | 12.6 | 5.02 | 88.4 | 0.057 | 0.82 |
| Standard deviation | 0.22 | 1.0 | 0.7 | 0.05 | 5.2 | 0.004 | 0.01 |
| M08 | 2.16 | 82.3 | 12.7 | 5.45 | 77.0 | 0.071 | 0.75 |
| Standard deviation | 0.24 | 0.7 | 0.7 | 0.23 | 1.6 | 0.002 | 0.00 |
| **M09** | **1.21** | **80.3** | **13.0** | **6.90** | **116.6** | **0.059** | **0.74** |
| Standard deviation | 0.02 | 0.1 | 0.5 | 0.19 | 7.0 | 0.002 | 0.03 |
| M10 | 2.07 | 78.3 | 14.2 | 7.51 | 81.6 | 0.092 | 0.70 |
| Standard deviation | 0.10 | 1.4 | 1.3 | 0.15 | 1.8 | 0.003 | 0.00 |
| M11 | 2.08 | 67.2 | 25.1 | 7.20 | 57.2 | 0.135 | 0.83 |
| Standard deviation | 0.33 | 6.3 | 5.5 | 0.26 | 7.9 | 0.023 | 0.01 |
| M12 | 2.06 | 80.7 | 13.3 | 5.96 | 91.2 | 0.065 | 0.72 |
| Standard deviation | 0.07 | 0.2 | 0.3 | 0.06 | 1.3 | 0.000 | 0.01 |
| M13 | 1.87 | 81.0 | 13.4 | 5.92 | 82.4 | 0.075 | 0.63 |
| Standard deviation | 0.11 | 0.6 | 1.0 | 0.18 | 10.4 | 0.008 | 0.02 |
| **M14** | **2.36** | **71.5** | **20.2** | **8.71** | **62.1** | **0.141** | **0.79** |
| Standard deviation | 0.03 | 1.7 | 1.0 | 0.43 | 1.2 | 0.009 | 0.01 |

| 1. Parenchyma cell size | | | | |  |
| --- | --- | --- | --- | --- | --- |
|  | Middle parenchyma cell diameter (µm) | Middle parenchyma cell diameter standard deviation (µm) | Parenchyma near the rind cell diameter (µm) | Parenchyma near the rind cell diameter standard deviation (µm) | |
| Abbreviation | Pm(Cd) | Pm(Cs) | Pr(Cd) | Pr(Cs) | |
| **M01** | **70.01** | **27.92** | **57.93** | **31.57** | |
| Standard deviation | 0.20 | 0.58 | 0.88 | 0.98 | |
| M02 | 79.71 | 29.37 | 54.19 | 30.13 | |
| Standard deviation | 3.43 | 1.79 | 2.72 | 0.30 | |
| M03 | 68.65 | 25.34 | 53.82 | 29.68 | |
| Standard deviation | 0.64 | 0.63 | 1.60 | 1.62 | |
| **M04** | **81.84** | **31.59** | **54.91** | **30.64** | |
| Standard deviation | 0.66 | 0.20 | 0.23 | 0.45 | |
| M05 | 64.11 | 27.08 | 51.91 | 32.55 | |
| Standard deviation | 2.11 | 0.90 | 0.92 | 1.34 | |
| M06 | 74.75 | 30.99 | 69.05 | 31.17 | |
| Standard deviation | 0.55 | 0.10 | 0.55 | 1.15 | |
| M07 | 71.99 | 29.05 | 56.62 | 30.42 | |
| Standard deviation | 3.40 | 0.82 | 0.89 | 1.00 | |
| M08 | 78.80 | 26.95 | 59.71 | 29.82 | |
| Standard deviation | 0.54 | 0.35 | 1.00 | 0.86 | |
| **M09** | **65.54** | **29.12** | **45.99** | **27.39** | |
| Standard deviation | 0.79 | 0.93 | 0.65 | 0.56 | |
| M10 | 76.79 | 26.98 | 57.65 | 33.75 | |
| Standard deviation | 1.53 | 0.67 | 0.46 | 1.27 | |
| M11 | 61.94 | 27.20 | 53.34 | 32.51 | |
| Standard deviation | 0.42 | 0.66 | 2.35 | 1.39 | |
| M12 | 61.29 | 28.87 | 48.97 | 31.45 | |
| Standard deviation | 2.65 | 0.49 | 1.66 | 0.67 | |
| M13 | 72.26 | 28.66 | 53.27 | 33.67 | |
| Standard deviation | 2.99 | 0.23 | 2.94 | 0.85 | |
| **M14** | **64.21** | **29.41** | **56.15** | **34.88** | |
| Standard deviation | 0.62 | 0.56 | 1.72 | 1.27 | |

|  | 1. Parenchyma cell wall density | |
| --- | --- | --- |
|  | Middle parenchyma Cell wall density (%) | Parenchyma near the rind Cell wall density (%) |
| Abbreviation | Pm(CD) | Pr(CD) |
| **M01** | **4.29** | **5.18** |
| Standard deviation | 0.01 | 0.08 |
| M02 | 3.78 | 5.58 |
| Standard deviation | 0.16 | 0.28 |
| M03 | 4.37 | 5.59 |
| Standard deviation | 0.04 | 0.16 |
| **M04** | **3.67** | **5.46** |
| Standard deviation | 0.03 | 0.02 |
| M05 | 4.70 | 5.79 |
| Standard deviation | 0.15 | 0.10 |
| M06 | 4.01 | 4.35 |
| Standard deviation | 0.02 | 0.04 |
| M07 | 4.20 | 5.30 |
| Standard deviation | 0.20 | 0.08 |
| M08 | 3.81 | 5.03 |
| Standard deviation | 0.03 | 0.09 |
| **M09** | **4.58** | **6.53** |
| Standard deviation | 0.06 | 0.09 |
| M10 | 3.91 | 5.21 |
| Standard deviation | 0.08 | 0.04 |
| M11 | 4.84 | 5.66 |
| Standard deviation | 0.03 | 0.25 |
| M12 | 4.92 | 6.15 |
| Standard deviation | 0.21 | 0.21 |
| M13 | 4.17 | 5.68 |
| Standard deviation | 0.17 | 0.32 |
| **M14** | **4.67** | **5.36** |
| Standard deviation | 0.05 | 0.16 |

| 1. Tissue cell wall proportion | | | | | |
| --- | --- | --- | --- | --- | --- |
|  | Total cell wall area (%) of the stem area | Middle parenchyma Cell wall area (%) of the total cell wall | Parenchyma near the rind Cell wall area (%) of the total cell wall | Rind cell wall area (%) of the total cell wall | Vascular bundle Cell wall area (%) of the total cell wall |
| Abbreviation | CW(T) | Pm(Cw) | Pr(Cw) | Ri(Cw) | Vb(Cw) |
| **M01** | **32.7** | **6.33** | **3.92** | **65.3** | **25.3** |
| Standard deviation | 2.0 | 0.63 | 0.22 | 2.0 | 1.2 |
| M02 | 30.3 | 6.18 | 4.74 | 64.0 | 26.2 |
| Standard deviation | 1.7 | 0.76 | 0.58 | 1.9 | 0.7 |
| M03 | 28.9 | 8.37 | 4.25 | 68.7 | 18.6 |
| Standard deviation | 1.1 | 0.51 | 0.27 | 1.7 | 1.0 |
| **M04** | **23.1** | **8.79** | **6.30** | **58.6** | **27.0** |
| Standard deviation | 0.5 | 0.28 | 0.15 | 0.8 | 0.7 |
| M05 | 30.9 | 7.72 | 4.42 | 59.4 | 29.1 |
| Standard deviation | 1.9 | 0.44 | 0.43 | 2.6 | 1.8 |
| M06 | 37.7 | 4.63 | 2.31 | 69.7 | 23.3 |
| Standard deviation | 0.5 | 0.01 | 0.06 | 0.8 | 0.8 |
| M07 | 21.4 | 10.70 | 6.97 | 60.6 | 23.6 |
| Standard deviation | 0.8 | 0.60 | 0.51 | 1.0 | 0.9 |
| M08 | 21.6 | 10.85 | 4.99 | 59.8 | 25.2 |
| Standard deviation | 0.9 | 0.80 | 0.21 | 1.0 | 0.3 |
| **M09** | **24.1** | **10.07** | **7.37** | **56.4** | **28.6** |
| Standard deviation | 0.3 | 0.14 | 0.11 | 1.2 | 1.1 |
| M10 | 25.0 | 9.15 | 4.24 | 58.2 | 30.2 |
| Standard deviation | 1.4 | 0.63 | 0.16 | 2.0 | 1.3 |
| M11 | 35.7 | 7.41 | 3.30 | 68.3 | 21.3 |
| Standard deviation | 5.4 | 2.09 | 0.51 | 5.2 | 2.6 |
| M12 | 23.5 | 12.42 | 5.55 | 58.6 | 25.3 |
| Standard deviation | 0.3 | 0.46 | 0.07 | 0.5 | 0.6 |
| M13 | 23.0 | 10.69 | 5.55 | 59.7 | 25.7 |
| Standard deviation | 0.7 | 0.70 | 0.61 | 2.5 | 1.2 |
| **M14** | **32.4** | **7.68** | **3.07** | **63.2** | **26.8** |
| Standard deviation | 1.3 | 0.49 | 0.08 | 0.7 | 0.6 |
